# Recent Advances toward Enhanced Photocatalytic Proprieties of BiFeO_3_-Based Materials

**DOI:** 10.3390/nano14010051

**Published:** 2023-12-23

**Authors:** Yassine Nassereddine, Manal Benyoussef, Bouchra Asbani, Mimoun El Marssi, Mustapha Jouiad

**Affiliations:** Laboratory of Physics of Condensed Matter, University of Picardie Jules Verne, Scientific Pole, 33 Rue Saint-Leu, CEDEX 1, 80039 Amiens, France; yassine.nassereddine@u-picardie.fr (Y.N.); manal.benyoussef@u-picardie.fr (M.B.); bouchra.asbani@u-picardie.fr (B.A.); mimoun.elmarssi@u-picardie.fr (M.E.M.)

**Keywords:** BiFeO_3_-based materials, substitutions, doping, heterostructures, photocatalysis, photodegradation

## Abstract

Owing to their remarkable success in photocatalytic applications, multiferroic BiFeO_3_ and its derivatives have gained a highly promising position as electrode materials for future developments of efficient catalysts. In addition to their appropriate band gaps, these materials exhibit inherent intrinsic polarizations enabling efficient charge carrier separation and their high mobility without the need for additional co-catalysts. Here, we review the existing strategies for enhancing the photocatalytic performances of BiFeO_3_-based materials and we describe the physico-chemical properties at the origin of their exceptional photocatalytic behavior. A special focus is paid to the degradation of organic pollutants and water splitting, both driven through photocatalysis to unveil the correlation between BiFeO_3_ size, substitution, and doping on the one hand and the photocatalytic performances on the other hand. Finally, we provide practical recommendations for future developments of high-performing BiFeO_3_-based electrodes.

## 1. Introduction

The global energy crisis and climate change are driven by the continuously growing world population and industrialization, which are heavily weighing on the future of human well-being and safety [1]. Nowadays, although fossil fuels represent more than 80% of the world’s energy consumption [2], an increased consciousness among policymakers and the population is emerging for taking urgent measures and actions to cope with CO_2_ footprints. Alternative clean energy sources hold strong potential to overcome environmental issues by limiting the use of fossil fuels. Hydrogen (H_2_) is a promising energy carrier and green fuel source capable of replacing the energy generated from non-renewable resources such as oil, coal, and natural gas [3,4,5,6,7,8,9,10,11,12,13]. H_2_ is produced using a variety of methods, including water thermolysis, electrolysis, methane-steam reforming, biofuel reforming, gasification, plasma arc decomposition, and the thermochemical conversion of biomass [14,15,16]. Recently, water splitting using solar irradiation has emerged as a promising process for H_2_ production, attracting considerable interest in the scientific and industrial communities. Solar-induced water splitting (WS) techniques that are classified include photocatalysis (PC), photoelectrochemical (PEC), and photovoltaic-photoelectrochemical (PV-PEC) systems [17,18,19]. PC systems correspond to a simple and low-cost process in which photocatalyst particles are dispersed in water for H_2_ production under light irradiation. Nevertheless, PC systems exhibit very low solar-to-H_2_ efficiency (STH), requiring highly selective materials for separating the produced H_2_ and O_2_ gases [20,21,22,23]. PV-PEC systems are very effective for WS owing to their high overall efficiency [24]. However, their high cost and the need for advanced technical expertise constitute two major drawbacks [25]. PEC systems are the most promising techniques for producing H_2_ in an easy, affordable, and sustainable way [26,27,28]. A typical PEC WS system is composed of semiconducting photoelectrodes, an electrolyte, a counter electrode, and a light source [29]. It features the combination of solar energy and water electrolysis in a single reactor with an STH efficiency of up to 12.4%. In addition, a PEC system does not require gas separation since H_2_ and O_2_ are already produced in two spatially distinct compartments [30]. It is worth noting that three essential criteria must be established for an effective and sustainable PEC. First, the semiconductor electrode material must exhibit a suitable band gap (~1.8 eV) [31], which is essential for achieving good band edge alignment concerning water redox potentials. Unfortunately, the best known good WS photocatalyst has a wide band gap energy that restricts light absorption, thus leading to weak photocatalytic performance [32,33]. Second, the semiconductor must be photo-corrosion resistant during its exposure to aqueous solutions and irradiation to avoid the formation of defects and the alteration of its photocatalytic properties while in use, which can lead to lower efficiency and a shorter lifespan [34,35]. Third, the charge transfer and separation in the semiconductor must be favorable and not hindered by the semiconductor’s surface overpotential. Recall that free electrons generated in the conduction band (CB) of the semiconductor (photoanode) will travel to the photocathode to drive the water reduction and hydrogen evolution reaction (HER). Holes generated in the valence band (VB) of the photoanodes will induce an oxygen evolution reaction (OER). Therefore, the ease of charge transfer and the separation of electron holes in the semiconductor material are crucial for improving the overall efficiency of the PEC WS process [36]. To date, several metal oxides have been used as semiconductors in PEC cells for WS, such as TiO_2_, ZnO, α-Fe_2_O_3_, and WO_3_ [37,38,39,40]. TiO_2_ is among the most widely used materials owing to its advantageous properties, including high stability and wide band gap (~3.2 eV) [41]. However, its high electron–hole recombination rate, high cost, and low visible light absorption limit its use in PEC-WS cells [42,43].

Recently, oxide perovskite materials (PMs) have attracted great attention for their use in PEC WS owing to their high photocatalytic properties, broadband absorption, low cost, facile synthesis, and well-controlled composition and morphology [44,45]. Moreover, their ferroelectric properties could promote their photocatalytic activity [46,47] as the intrinsic polarization in ferroelectric materials significantly contributes to reducing losses due to electron–hole recombination and back reactions in the system, which will increase the STH efficiency [48]. Additionally, the use of ferroelectric materials with a high dielectric constant can lead to a further increase in the charge separation efficiency, notably enhancing the photocatalytic activity [49]. However, more research is needed to fully understand the relationship between ferroelectric properties and photocatalytic activity.

Nowadays, the increasing global need for water resources coupled with their dwindling availability has emerged as a significant global concern [50]. While recycling wastewater has been suggested as a solution to address water scarcity, the presence of harmful organic substances like pesticides, phenols, and organic dyes in wastewater has raised multiple concerns regarding its environmental impact [51]. Practical solutions and strategies have been adopted to achieve more sustainable water resources. Since solar energy is one of the most accessible renewable energy sources, it makes sense to use it in combating waste degradation by synthesizing materials that can be put to direct use [52]. The development of an innovative technology, known as waste degradation through photocatalysis, is currently underway to address the issue of harmful pollutants. This process involves harnessing the power of light to initiate a chemical reaction in a photocatalyst material resulting in the conversion of these pollutants into less toxic or non-toxic substances. When exposed to light, electron–hole pairs are generated by the photocatalyst, which can serve as potent oxidizing or reducing agents. These electron–hole pairs can subsequently undergo reactions with water or oxygen molecules, leading to the creation of highly reactive oxygen species (ROS) such as hydroxyl radicals (•OH), superoxide radicals (•O_2_^−^), and hydrogen peroxide (H_2_O_2_) [53]. These ROS can be employed to facilitate the breakdown of organic and inorganic pollutants in waste materials, ultimately transforming them into simpler and less harmful compounds. Nonetheless, a recent development has sparked significant interest in a novel set of materials categorized within the perovskite structure class, particularly for their potential applications in photodegradation [54].

For instance, PbTiO_3_ is regarded as a potential material for photocatalytic applications due to its promising properties since its internal electric field could ensure an effective charge separation and prevent electron–hole recombination [55]. However, lead is a toxic element with known environmental effects [56]. In this sense, lead-free BiFeO_3_ (BFO) could be considered a potential multiferroic material with a high spontaneous polarization value of P~90 μC·cm^−2^ [57,58]. BFO has been widely used in various applications, including organics degradation, air purification, and H_2_ production (i.e., as a photoanode) [59,60,61]. In addition, BFO exhibits a high absorption coefficient in the visible region and relative stability under photocatalytic conditions [62]. Yet, the band alignment of BFO needs to be tuned to the water redox potentials to increase the STH [63]. To enhance the photocatalytic activity of BFO, several strategies were employed, such as doping/co-doping, size control, surface modification, co-catalysts, and heterostructures [64,65,66,67,68].

This review covers a large spectrum of strategies used to enhance the photocatalytic performances of BFO-based materials made by alloying, substitution, doping, and heterostructures. It aims to highlight efficient routes for improving the photocatalytic properties of BFO-based materials and to provide practical recommendations.

## 2. Bismuth Ferrite

**Figure 1:**
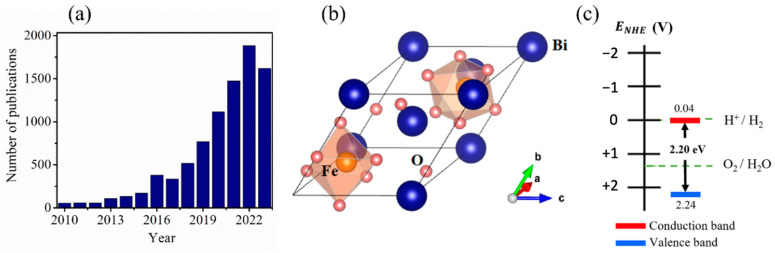
(**a**) Publications related to a BFO photocatalyst from the period between 2010 and 2023, (**b**) BFO crystal structure (Rhombohedral R3c) with correspondence of Bi, A-Site, and Fe, B-site and (**c**) BFO band alignment concerning water redox potentials (reproduced with permission from [76]).

Recent years have witnessed a spurring interest in BFO as a highly promising photocatalyst material owing to its outstanding crystalline structure and ferroelectric properties. This interest is reflected by the remarkably increasing number of studies published in BFO-based materials since 2010, as shown in Figure 1a. BFO has a rhombohedral R3c crystal structure at room temperature (Figure 1b) with unique multiferroic characteristics. Namely, it exhibits both ferroelectric Curie temperature (T_C_ = 830 °C) and antiferromagnetic Neel temperature (T_N_ = 370 °C) properties, demonstrating ferroelectric coupling and magnetoelectric effects [69,70]. These properties enhance its capacity to efficiently separate charge carriers, which is essential to make BFO catalytically active in ultrasonic and magnetic fields [71]. They also contribute to its exceptional magnetic recycling properties and chemical stability. Moreover, BFO exhibits a relatively narrow band gap in the visible light spectrum in the range between 2.0 eV and 2.7 eV [72,73,74]. These characteristics drive superior photocatalytic performances compared with various other perovskite materials. In our previous work, we used density functional theory to show the strong hybridization between Bi 6s, Fe 3d, and O 2p orbitals [72]. It is worth noting that the strong hybridization between Bi 6s and O 2p orbitals makes BFO highly oxidizing and promotes outstanding charge mobility [75].

Apart from these characteristics, the key property required in a photocatalyst material for efficient water splitting is the appropriate band alignment with water’s redox potentials. It was previously demonstrated that BFO presents a good alignment with the oxidation potential of water, suggesting an effective O_2_ evolution reaction (OER) [76]. Nonetheless, lowering its conduction band minimum (E_CB_) below the EH^+^/H_2_ energy remains needed to enable an efficient H_2_ evolution reaction (HER) (Figure 1c) [76]. This requirement has driven numerous research efforts for enhancing BFO photocatalytic characteristics to achieve more efficient and substantial H_2_ production. The strategies adopted for this purpose include investigating the effects of the elaboration technique, dimensionality/size, doping and substitution, and heterostructure [77]. Next, we will detail some of these strategies and compare their efficiencies.

### 2.1. Size Effect

**Figure 2:**
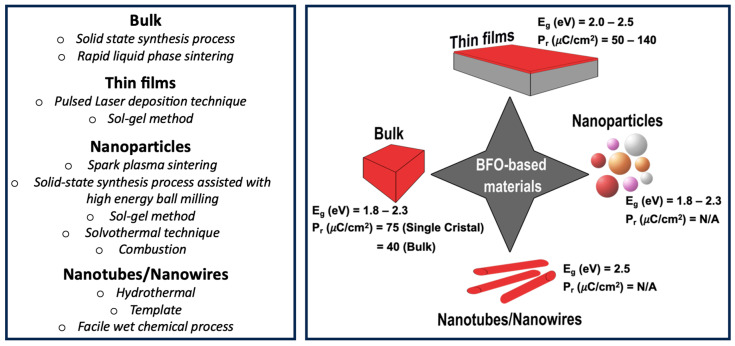
Strategies commonly adopted to control the size and morphology of BFO-based materials and their physical properties with respect to their dimensions.

The physical properties of multiferroic BFO-based materials are strongly dependent on a variety of parameters, such as grain and particle sizes, dopants, and substitutions. These parameters, usually affected by the fabrication method, could be tailored toward desired properties for targeted applications. Extensive research has been reported on the particle size effect on BFO’s physical properties such as its dielectric constant (ԑ_r_) and remanent polarization (P_r_), as summarized in Table 1 and illustrated in Figure 2. The dielectric permittivity (ɛr) and remanent polarization (Pr) are critical parameters in ferroelectric materials, playing an important role in photocatalytic applications by facilitating effective charge separation and mobility [78]. Note that ɛr defines the material’s electrical polarizability, while Pr indicates the material’s ability to retain polarization even without an external electric field. For ferroelectric materials based on photocatalysis, these characteristics contribute to the creation of a built-in electric field promoting electron-and-hole separation and enabling them to migrate towards opposite polarities. This additional driving force is essential to prevent e-h recombination and back reactions within the system, thereby significantly enhancing the photocatalytic performances of the materials [79]. Large P_r_ values (~40 μC·cm^−2^) were reported for bulk BFO ceramics [80], while single crystals grown using the flow method exhibited high P_r_ values (~75 μC·cm^−2^) with low leakage currents [81]. Similarly, highly resistive single-phase ferro electromagnetic BFO ceramics (particle size of 0.5–1 µm) were fabricated using a rapid liquid phase sintering technique at 880 °C for 450 s. These ceramics exhibited saturated ferroelectric hysteresis loops indicating a remanent polarization of ~8.9 µC·cm^−2^ at room temperature [82].

The elaboration of BFO ceramics (grain size of ~200 nm) exhibiting dielectric constants and of the remanent polarization of 25 (RT/10^2^ Hz) and 7.5 μC·cm^−2^, respectively, have been reported using a hybrid fabrication method involving spark plasma sintering and conventional solid-state synthesis techniques associated with high energy milling [83]. Moreover, using glycine as a chelating agent, a high-purity BFO single-phase can be obtained with a reduced calcination time. The resulting BFO powders with a particle size of ~39.7 nm exhibited a high dielectric constant of ~1118 at 42 Hz at room temperature (RT) [84]. Furthermore, BFO nanoparticles (NPs) with an average size of ~12 nm synthesized through the sol–gel method, were reported with a dielectric constant and a remanent polarization of ~84.53 (RT/10^2^ Hz) and ~8.2 µC·cm^−2^, respectively [85]. Solvothermal techniques, using hexa-methylenetetramine as a precipitating agent with different concentrations, were employed to synthesize nanometric BFO powder with a very high dielectric constant value of ~4000 (RT/10^2^ Hz) and a high remanent polarization of ~6.65 µC·cm^−2^ [86].

### 2.2. Doping and Substitution Effects

Doping and substitution are two common methods used to modify the physical properties of semiconductors (Table 2). Doping involves introducing impurities into the semiconductor crystal lattice, while substitution involves replacing some of the atoms in the lattice with different atoms. Y. Du et al. reported the synthesis of multiferroic micro-particles Bi_1−x_La_x_FeO_3_ using a hydrothermal technique [90]. After doping with La, the Bi_1−x_La_x_FeO_3_ sample exhibited an increase in its dielectric constant, with the highest value 225 (at RT/10^2^ Hz) being observed in the sample with x = 0.2 and a particle size of 10 µm in both low and high frequency ranges at room temperature. Moreover, a comparative study of La^3+^-doped multiferroic BFO (LBFO) showed that BFO with a high purity could be obtained using the sol–gel (SG) synthesis process at relatively lower temperatures, whereas powder with a minor quantity of the secondary Bi_25_FeO_40_ phase is obtained using the solid-state (SS) method. The sol–gel method was used to prepare a single-phase LBFO with a smaller particle size of approximately 0.4 μm. When compared with LBFO prepared using the solid-state reaction method, the SG-prepared LBFO demonstrated a significantly higher dielectric constant value on the order of 50,000 (at RT/10^2^ Hz) [91]. Furthermore, Zhang et al. effectively fabricated high-quality Bi_1−x_La_x_FeO_3_ thin films on fluorine-doped tin oxide (FTO)/glass substrates through a sol–gel methodology employing a spin-coating technique. Their research findings indicate that the inclusion of La^3+^ ions resulted in a notable decrease in the concentration of Fe^2+^ ions and O_2_ vacancies within the material. This led to a significantly large remnant polarization value (P_r_ = 140.2 μC·cm^−2^) and a high dielectric constant (ε_r_ = 161.77 at RT/10^2^ Hz) for the thin film Bi_0.98_La_0.02_FeO_3_ with an average grain size of 90 nm [92]. Sheoran et al. reported the synthesis of Yttrium (Y^3+^)-substituted BFO (Bi_1−x_Y_x_FeO_3_) nanostructures through the sol–gel pursued auto-combustion route. The study results indicated that the sample with x = 0.2 exhibited a maximal dielectric value of 500 at RT/10^2^ Hz, and a P_r_ value of 16 μC·cm^−2^, which can be attributed to its high density of space-charge polarization as a result of its small grain size of 41 nm [93]. In their study, Dhir et al. described the synthesis of Gd-doped BFO nanoparticles using the sol–gel method. The incorporation of Gd^3+^ ions was found to have a positive impact on both the magnetic and electric properties of the material. Notably, the reduction in particle size (16 nm) for x = 0.15 led to further improvement in the dielectric constant (2193 at RT/10^2^ Hz) and remanent polarization (7 μC·cm^−2^) values [94]. In addition, the influence of Yb doping on the electrical characteristics of BFO fabricated using the hydrothermal method was examined. BFO doped with 3% Yb displayed the greatest remanent polarization (P_r_) value of 0.37 μC·cm^−2^. However, the dielectric measurements revealed that the introduction of Yb into the perovskite structure of BFO (18–29 nm) improved the formation of M-O-M bonds, resulting in the highest dielectric constant value of 150 (at RT/10^2^ Hz) in BYbFO with x = 0.1 [95]. Using the same synthesis technique, the dielectric properties of barium-doped BFO nanoparticles (Bi_1−x_Ba_x_FeO_3_) have been investigated. The obtained dielectric constant value for Bi_1−x_Ba_x_FeO_3_ nanoparticles (x = 0.015) of 125 (at RT/10^2^ Hz) was found to be higher than that of pure BFO. This observation can be attributed to the smaller grain size (57.1 nm) and the increased density of defects, such as O_2_ vacancies, resulting in a substantial space-charge polarization [96]. Furthermore, Mazumder et al. reported the effect of Pb-doping on the dielectric properties of BFO prepared through a straightforward simultaneous precipitation technique which was subsequently followed using a traditional sintering process. The measured dielectric constant and remanent polarization values for Bi_1−x_Pb_x_FeO_3_ (x = 0.03) are 2500 (at RT/10^2^ Hz) and 0.75 μC·cm^−2^, respectively [97]. To study the influence of rare earth element (La^3+^, Eu^3+^, Er^3+^) doping on BFO electrical properties, the microwave-assisted modification of solution combustion synthesis was used to fabricate nanocrystalline (18–28 nm) BFO rare earth-doped powders. It was discovered that the crystal cells of the obtained materials were significantly distorted through rare earth doping, which led to the formation of mixed rhombohedral/orthorhombic crystal structures with decreased lengths of Bi-O and Fe-O bonds and a decreasing radius size of doping ions. As a result, the dielectric constant of the materials was enhanced. The highest dielectric constant value of 150 (at RT/10^2^ Hz) was found in Bi_0.9_Eu_0.1_FeO_3_ [98]. Likewise, Rani et al. investigated the effect of doping Er^3+^ into BFO on its dielectric properties. The Er-doped BFO samples exhibited a marked improvement in their dielectric constant values (500 at RT/10^2^ Hz for x = 0.15) that can be linked to a decrease in both O_2_ vacancies and leakage current [99]. Conversely, different studies have been conducted on the effect of doping and substitution of the BFO B-site on its dielectric properties. The Ti-doping effect on the dielectric properties of BFO nanoparticles synthesized using the solvothermal method has been reported. A doping rate of 5% Ti had a considerable effect on increasing the dielectric constant, reaching 1000 at RT/10^2^ Hz with a particle size of 695 nm [100]. In addition, Kathirvel et al. reported Zr-doped BFO nanostructures fabricated through the hydrothermal method. At a dopant concentration of 2.5% Zr with a particle size of 46 nm, the dielectric constant was found to be 366 at RT/10^3^ Hz. The observed increase in the dielectric constant can be attributed to the reduction of Fe^3+^ ions and an increase in O_2_ vacancies [101]. Tuning the dielectric properties has been reported through doping BFO with Ni. The matrix Ni-doped BFO (0 ≤ x ≤ 0.07) was prepared using a cost-effective conventional sol–gel technique. Except for the 3% doped sample, the Ni-doped BFO material demonstrated enhanced dielectric properties, as evidenced through an increase in the dielectric constant reaching 2000 (at RT/10^2^ Hz) for 1% Ni dopant [102]. In a comparative study, Saxena et al. reported the dielectric properties of rare-earth ion substitution at dual sites within the BFO crystal. The high dielectric constant was found for the Bi_0.9_La_0.1_Fe_0.95_Ni_0.05_O_3_ (BLFNO) composition with a corresponding value of 2083. However, the remanent polarization decreased dramatically as the doping rate increased, going from 17.04 µC·cm^−2^ for BFO to 0.64 µC·cm^−2^ for BLFNO [103]. Another study was performed on the impact of the co-doping of BFO with Ba and Nb on the physical properties. It was found that the dielectric constant at higher frequencies increases (with the value of 115 RT/10^2^ Hz for 10BaNb) with the doping level, while the dielectric loss decreases. This can be attributed to a reduction in defect centers achieved through co-doping, which has a considerable effect on the particle size (27 nm for 10BaNb). Increasing the doping level improves the saturation level of the ferroelectric loop and increases the remnant polarization, which rises from 1.28 μC·cm^−2^ in the 5% Ba-doped sample to 3.24 μC·cm^−2^ in the 10% Ba–Nb co-doped sample [104]. Xu et al. studied the electrical properties of Li/Nb co-doping BFO (BiFe_1−x_(Li_0.5_Nb_0.5_)_x_O_3_) fabricated using traditional ceramic sintering techniques. A high dielectric constant value of 1050 was obtained for the sample with x = 0.01. Likewise, the addition of a small amount of Li/Nb co-doping to BFO ceramics led to enhanced electrical properties, as evidenced through the improvements in the remnant polarization and the ferroelectric P-E loop shape. The observed improvements are thought to be related to a decrease in the concentration of O_2_ vacancies and a decrease in the formation of Fe^2+^ ions compared with the results obtained with pure BFO [105].

**Figure 3:**
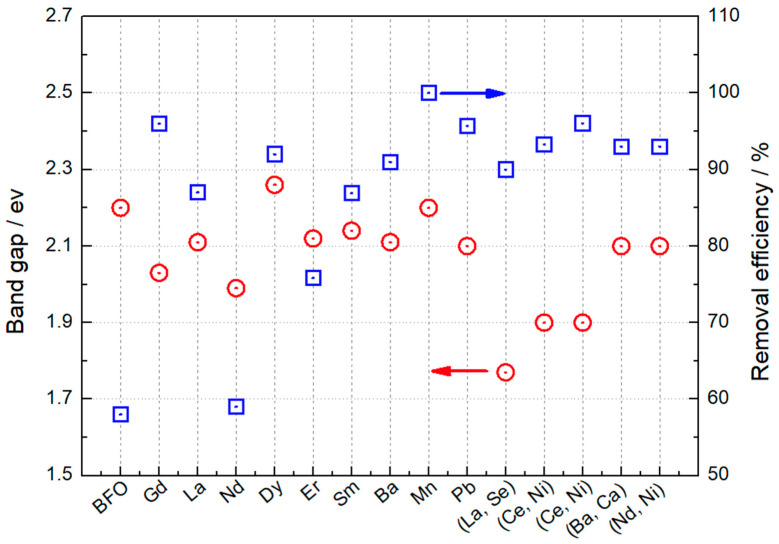
Effect of doping and co-doping on the band gap energy and pollutant removal efficiency of BFO under visible light.

Designing efficient photocatalytic materials based on BFO for photocatalysis application is challenging. Although BFO-based photocatalysts have demonstrated significant promise in the degradation of organic contaminants, their practical application is limited by unresolved issues such as large band gaps, high recombination rates of photogenerated electrons and holes, and a low separation rate of the photogenerated carriers. Therefore, to maximize solar energy harvesting and boost the adsorption of photodegraded organic compounds, it is imperative to develop photocatalysts with a high selective adsorption capacity and a suitable semiconducting band gap that can be achieved through doping. This approach brings about the basic properties of the tunable surface that depend on the nature and composition of the dopants. It is crucial to comprehend how doping affects photocatalyst qualities in order to select the appropriate element doping. In order to achieve more visible light harvesting, the introduced dopants are intended to (i) improve the surface and interface properties; (ii) modify the large band gap and electronic structure; and (iii) improve each step in the charging kinetics to reduce the recombination of photogenerated carriers. Figure 3 uses a scheme to sum up the effects of doping and co-doping on the band gap energy and removal efficiency of BFO material under visible light.

### 2.3. Effect of BFO-Based Heterostructures

Recent years have witnessed extensive research on heterostructure-based oxide perovskites owing to their exceptional properties and potential applications. The combination of oxide perovskites can significantly affect their physical properties. These heterostructures could exhibit unique electronic, magnetic, and optical behaviors that are not often found in their constituent counterparts. The interfaces generated by the association of perovskites usually induce strain, charge transfer, and defect generation, thereby prompting modifications in the electronic structure and transport properties. Hence, all these properties can be engineered by controlling the composition, thickness, and orientation of the constituent layers. It has been shown that the BFO heterostructures made with the combination of SrTiO_3_, La_0.7_Sr_0.3_MnO_3_, and BaTiO_3_ exhibit improved electric and dielectric properties.

Sen et al. investigated the multifaceted properties of (BiFeO_3_)_0.6_(CaTiO_3_)_0.4_ prepared following a solid solution approach. The structural deformation from the pure BFO’s rhombohedral structure to an orthorhombic phase was indicated through Rietveld refinement. At RT/10^2^ Hz, the dielectric constant was improved with a high value of 1050 [108].

In another work, a solid solution of 0.8Bi_1−x_Nd_x_FeO_3_-0.2PbTiO_3_ (BNFPT)_x_ was prepared using the conventional solid-state reaction process, and the effect of Nd^3+^ substitution on the electric and dielectric properties was examined. The coexistence of tetragonal and rhombohedral structures was established by X-ray diffraction and Raman spectroscopic analyses of the samples. The introduction of Nd^3+^ into the 0.8BiFeO_3_-0.2PbTiO_3_ compound was found to enhance the dielectric constant (1625 at RT/10^2^ Hz for x = 0.05) and reduce the dielectric loss [109].

Ren et al. studied the Mn-doped 0.5BiFeO_3_-0.5SrTiO_3_ ceramics in three different ways: as an additive, by substituting Fe sites, and by substituting Ti sites. The findings demonstrated that the dielectric and ferroelectric properties of the ceramics are significantly influenced by how Mn is inserted. The authors reported that using Mn either as an additive or a substituent of the Fe site can lead to a reduction in the dielectric loss and an enhancement in the dielectric breakdown field. However, the Mn substitution of the Ti site can negatively impact the dielectric behavior but encourage grain growth. The reduction of Fe and O_2_ vacancies can be effectively addressed by both adding Mn and substituting the Fe site. Moreover, substituting the Ti site with Mn can lead to the creation of more O_2_ vacancies. At room temperature, it was found that samples with 1% (mole) Mn-doped BFO-STO exhibit a remnant polarization of 6 μC·cm^−2^ and a dielectric constant of 720 at 10^2^ Hz [110]. A high dielectric constant value (4300 at RT/10^2^ Hz) was obtained for BiFeO_3_-BaTiO_3_ solid solution ceramics fabricated using microwave sintering (MWS) and traditional sintering (CS) techniques for a solid-state reaction [111].

Furthermore, the effect of the thermal quenching and sintering temperature on the physical properties of sol–gel-synthesized BiFeO_3_-xPbTiO_3_ nano-ceramics was investigated showing a very large remnant polarization of 95 μC·cm^−2^ and a dielectric constant value of 587 at (RT/10^2^ Hz) for BF-34PT [112]. Recently, more complex systems of three perovskite oxides, one of which is BFO, have been developed. The solid-state reaction method was used to fabricate the ternary compound BiFeO_3_-BiCoO_3_-BaTiO_3_ (BFO-BCO-BT) and tuning the dielectric constant and ferroelectricity was investigated. The buildup of interfacial charges and the stabilization of the BFO-BT solid solution through the addition of BCO showed a large dielectric constant value of 2000 at RT/10^2^ Hz with a small particle size of 60 nm [113]. Zhang et al. studied the effect of Ta_2_O_5_-modified BiFeO_3_–BaTiO_3_–LaFeO_3_ solid solutions prepared using the solid-state reaction on the dielectric and multiferroic properties. The incorporation of Ta_2_O_5_ into ceramic samples resulted in enhanced dielectric properties, as evidenced by an increase in the dielectric constant from 829 for undoped ceramics to 1149 for x = 1.25 measured at RT/10^2^ Hz [114]. Table 3 summarizes the dielectric constant and polarization values of BFO-based heterostructures concerning grain/particle size.

## 3. BFO-Based Materials Photocatalytic Applications

### 3.1. Degradation of Organic Pollutants

Recently, TiO_2_ and ZnO have been extensively exploited for their various photocatalytic applications. However, their ability to absorb in the UV region (which comprises only 10% of the total sun radiation) and the challenge of their removal after treatment have hindered their usage as photocatalysts [119]. Therefore, developing a suitable catalyst working in the visible region is a currently pressing need. BFO, a room-temperature multiferroic material, constitutes an attractive candidate owing to its activity in the visible region and its magnetic behavior favoring an easy removal of the photocatalyst after treatment. In addition, its photocatalytic activity under visible light becomes prominent due to its narrow band gap of 2.1–2.7 eV, which is particularly important because visible light energy occupies about 48% of the total solar energy [120].

Recently, numerous works have been reported on BFO for the photocatalytic degradation of dyes such as methyl orange, methylene blue, and rhodamine B [121]. Mohan et al. were the first to report the activity of nanostructured BFO particles on the degradation of methylene blue (MB) under sunlight, demonstrating a 58% degradation efficiency after 240 min [122]. Doping is among the most important ways to efficiently enhance the photodegradation performance of BFO (Table 4) considering the wide scope of designs to alter both A- and B-sites. Previous studies have shown that doping offers extra photocatalytic advantages to reduce the bandgap and other photophysical properties of this functional oxide [123].

A large majority of earth metals, such as Gd, La, Nd, Dy, Er, and Sm, have been introduced as dopants into BFO nanostructures to investigate their photocatalytic properties [124,125,126,127,128]. This route has proved to be relatively more successful due to the 4f electron configurations of rare earth metals that facilitate the abruption of photogenerated electron–hole pairs. The band gap of some bismuth photocatalysts has been shown to decrease with rare earth element doping, which might increase the photocatalytic activities. It should be noted that substituting Bi^3+^ cations with rare earth ions that have smaller ionic radii than Bi^3+^ (1.03 Å), such as Dy^3+^ (0.912 Å), Gd^3+^ (0.938 Å), or Sm^3+^ (0.958 Å), is requisite in order to cause significant structural distortions in the BFO lattice for improved photocatalytic properties. The photocatalytic activities of Gd-doped (10%) BFO were found to considerably enhance its photocatalytic performance under simulated solar irradiation. Findings show that 10% Gd BFO photocatalyst degradation rates reach 80% and 79% for ciprofloxacin and levofloxacin, respectively [121]. Similar results reported the degradation activity of Gd-doped BFO photocatalysts for decomposing methylene blue and rhodamine B under visible light irradiation. It was found that Gd-doped (10%) BFO photocatalysts exhibit much higher photocatalytic activity than pure BFO. Gd-doped BFO decomposes 94% of methylene blue after 240 min and 94% of rhodamine B after 120 min [124]. The distinctive photocatalytic efficiency of Gd-doped (10%) BFO can be largely attributed to its excellent morphology and good crystallinity that facilitate improved light absorption and the effective separation of photogenerated charge carriers. These results illustrate the excellent photocatalytic activity of Gd-doped (10%) BFO, which can be employed in various applications related to environmental remediation. Considerably enhanced photocatalytic activity was also obtained by adding La doping to BFO nanoparticles [125]. In this case, approximately 87% higher degradation of the methylene blue was observed after 70 min under visible-light illumination. La-doped BFO presents better photocatalytic activity compared to undoped BFO nanoparticles, which could be ascribed to the increase in the recombination rate of holes and electrons in doped samples or to band gap variations. In a recent study, Dy-doped BFO was found to induce a high photocatalytic degradation of methylene blue (92%) achieved after 240 min under visible-light irradiation [126], which could be attributed to the reduced band gap energy and ferroelectric properties. Increasing Dy concentrations up to 15% mediated a charge transfer process through band bending in this composition that was associated with enhanced electrical domains. Likewise, Er-doped BFO was used as a photocatalyst for the photocatalytic removal of tetracycline hydrochloride (TC) under visible light [127]. The photocatalytic activities of Er-doped BFO for TC removal were much higher than those for BFO, where Er-3%-doped BFO samples achieved the highest photocatalytic TC-degradation efficiency of 75.8% after 180 min (~2.8 times higher than that of the BFO samples). The Er-3%-doped BFO photoelectrode manifested higher photocurrent intensity compared with BFO photoelectrodes, implying a much more efficient charge separation and a transfer with a longer charge lifespan of the photoinduced carriers, thus improving the photocatalytic performance. Notably, Er is a popular rare earth element for doping semiconductor photocatalysts owing to its unique transitions of Er intra-f electrons that to the sensitization of the photocatalyst to visible light. Chen et al. reported an enhancement in photocatalytic activity of Nd-doped BFO with the increase in Nd-doping concentrations when x = 0.2 (59% after 120 min) [128]. However, the photocatalytic activity was found to decrease with the further increase of the Nd-dopant concentration. The maximum photocatalytic activity of x = 0.2 was ascribed to the anomalously high dielectric constant at the morphotropic phase boundary, enlarging the width of the space-charge region. This phenomenon results from the increase in the defect sites in the lattice, which enhances the charge separation and reduces electron/hole–pair recombination rates. Nonetheless, higher doping concentrations produce more defect sites that convert to recombination centers. Another study shows that the photocatalytic activity of Sm-doped BFO was significantly affected by the Sm-doping content [129]. Compared to pure BFO, the Sm-doped BFO samples exhibited much higher photocatalytic activity, which was attributed to the enhanced visible-light absorption and the efficient separation of photogenerated electrons and holes derived from Sm-dopant trapping level. Moreover, the visible-light photodegradation of organic dyes using BFO doped with Ba, Mn, Co and Pb metal ions was studied. Soltani and Lee reported a complete photocatalytic degradation of toluene and benzene with 91% and 81% reductions after 50 min under visible-light irradiation for Ba-doped BFO [130]. The BFO nanoparticles doped with Ba exhibited a low band gap energy, high specific surface area, and high ferromagnetic properties, all contributing to the improvement of the photocatalytic performance. The findings showed that Ba-doped BFO exhibits a decreasing band gap energy with reduced O_2_ vacancies, which is related to the lattice distortion of the Ba-doped BFO nanoparticles. In fact, the growth of the particles is restricted, leading to an increasing specific surface area and a significant improvement of the photocatalytic activity.

Photocatalysis has been reported for the degradation of AR-85 under visible-light irradiation using Mn-doped (10%) BFO photocatalysts [119]. The photocatalytic activity was demonstrated at 100% degradation of the dye in only 50 min after light exposure, whereas the degradation time required for the undoped bismuth ferrite was much longer. Mn-doped (10%) BFO led to a decrease in particle size, while the band gap gradually decreased from 2.2 eV to 1.97 eV with an increasing Mn content. The greater photocatalytic activity in Mn-doped BFO compared with pristine BFO is associated with the efficient separation and migration of photogenerated charge carriers and the decreased recombination probability of electron/hole pairs derived from the Mn ion doping. In another study, the effect of co-doping on the B-site of BFO was investigated [131]. A remarkable photocatalytic performance was observed for co-doped BFO with a degradation rate of 93.79% after 2 h under light exposure. The results indicate that co-doping promoted the effective charge separation of the catalyst to enhance photocatalytic behavior, which was attributed to the reduction in the crystal size and the creation of O_2_ vacancies in the system due to co-doping. Hence, co-doping improves the position of BFO as a promising candidate for environmental remediation applications. Jaffari et al. reported the effect of a Pd-doped BFO catalyst for the degradation of malachite green dye and phenol from waste water [132]. Particularly, the 2 wt% Pd-BFO exhibited the best photoactivity (95.7% degradation) compared with pure BFO (72.3% degradation). The enhanced photoactivity could be credited to the appropriate Pd contents that enhanced the e^−^-trapping capacity, which was helpful in the generation and transmission of e^−^/h^+^ pairs. The charge carrier generation and separation/transfer are key factors in the photocatalytic process. Furthermore, the separation/transfer of e^−^/h^+^ pairs using Pd-doped BFO photocatalysts were investigated under the on/off circulation of 105 W of visible light using transient photocurrent measurements. Pd-BFO possessed the highest current intensity of 2.59 μA, which was 1.6 times higher than that of pure BFO. These results explicitly revealed that the loaded metallic Pd on the BFO surface would highly expedite the generation and separation/transfer of charge carriers, which validated the improved photocatalytic ability of Pd-doped BFO to degrade organic pollutants.

Meanwhile, the co-substitution of the BFO structure in both the A- and B-sites with (La, Se), (Ce, Ni), (Nd, Ni), and (Ba, Ca) have been used to improve the photocatalytic activity and visible light response of the material compared with the bulk BFO material. The substitution of elements at the A-site can help suppress bismuth volatilization, while the substitution of transition metals at the B-site can reduce the Fe valence fluctuations. These changes in the elemental composition and oxidation state can result in improved photocatalytic activity, greater stability, and longer lifespan for BFO photocatalysts. These doping strategies have been widely investigated, offering great potential for developing even more efficient BFO photocatalysts. In this context, the co-substitution of La in place of Bi as well as Se in place of Fe was studied to control the recombination and enhance the number of delocalized electrons [133]. The photodegradation activity of La- and Se-co-doped BFO was investigated under visible-light irradiation using Congo red as a model dye in an aqueous solution. The developed material exhibited excellent photocatalytic activities for model dye, catalyzing more than 90% of the dye in the first 30 min of exposure to visible light. Higher dye degradation activities for La- and Se-co-doped BFO can be attributed to the complete phase transition from rhombohedral to orthorhombic, which provides a favorable band gap (1.77 eV) and binding energies for the enhanced catalysis of dye species. The lower band gap provided easy electron availability upon exposure to incident radiation, while the sheet-type morphology ensured larger contact between the surface of the catalyst and the adsorbing species, resulting in an enhanced synergistic response and higher catalytic activities.

**Table 4:** Pure BFO- and doped BFO-based photocatalyst for dye degradation.

Photocatalyst	Doping Elements	Band Gap (ev)	Polluant	Degradation Time	Removal Efficiency	Refs.
BiFeO_3_		2.2 eV	Methylene Blue	240 min	58%	[122]
10% Gd-BiFeO_3_	Gd	1.95–1.18 eV	Ciprofloxacin	240 min	80%	[121]
10% Gd-BiFeO_3_	Gd	1.95–1.18 eV	Levofloxacin	240 min	79%	[121]
10% Gd-BiFeO_3_	Gd	2.38–2.29 eV	Methylene Blue	180 min	97%	[122]
10% Gd-BiFeO_3_	Gd	2.03–2.2 eV	Rhodamine B	240 min	96%	[124]
Bi_0.90_La_0.05_Ba_0.05_FeO_3_	La	2.02–2.11 eV	Methylene Blue	70 min	87%	[125]
Bi_0.8_Nd_0.2_FeO_3_	Nd	1.99 eV	Rhodamine B	120 min	59%	[128]
Bi_0.85_Dy_0.15_FeO_3_	Dy	2.35–2.26 eV	Methylene Blue	240 min	92%	[126]
Er3%-BFO	Er	2.12 eV	Tetracycline hydrochloride	180 min	75,8	[127]
Bi_0.97_Sm_0.03_FeO_3_	Sm	2.14 eV	Methyl orange	120 min	86.9%	[129]
Bi_0.93_Ba_0.07_FeO_3_	Ba	2.11–1.86 eV	Toluene	50 min	91%	[130]
Bi_0.93_Ba_0.07_FeO_3_	Ba	2.11–1.86 eV	Benzene	50 min	81%	[130]
10% Mn-doped BFO	Mn	2.2–1.97 eV	Acid red 85	60 min	100%	[119]
BiFe_0.925_Co_0.075_O_3_	Co		Acid Red 85	240 min	93.79%	[131]
0.2 wt% Pd-BFO	Pb	2.10 eV	Malachite green		95.7%	[132]
Bi_0.92_La_0.08_Fe_0.95_Se_0.5_O_3_	(La, Se)	1.77 eV	Congo Red	30 min	90%	[133]
Bi_0.92_Ce_0.08_Fe_0.92_Ni_0.08_O_3_	(Ce, Ni)	1.9 eV	Methylene Blue	90 min	93.29%	[134]
Bi_0.92_Ce_0.08_Fe_0.92_Ni_0.08_O_3_	(Ce, Ni)	1.9 eV	Rhodamine B	90 min	96.05%	[134]
Bi_0.9_Ba_0.05_Fe_0.95_Ca_0.05_O_3_	(Ba, Ca)	2.1 eV	Methylene Blue	90 min	93%	[125]
Bi_0.95_Nd_0.05_Fe_0.97_Ni_0.03_O_3_	(Nd, Ni)	2.1 eV	Methylene Blue	90 min	93%	[135]
BiFeO_3_/BiVO_4_	-	2.7 eV	Tetracycline	90 min	95%	[136]
BiFeO_3_/MoS_2_	-	1.8 eV	Rhodamine B	200 min	89%	[137]
Ag/BiFeO_3_	-	2.2 eV	Methyl orange	120 min	96%	[138]
BiFeO_3_/rGO	-	1.9 eV	Methylene Blue	300 min	98%	[139]
Cu_2_O/BiFeO_3_	-	2/2.1 eV	Tetracycline	120 min	98%	[140]
BiFeO_3_/Bi_2_Fe_4_O_9_	-	2.2/1.9 eV	O-chlorophenol	240 min	95%	[141]
SnO_2_/BiFeO_3_	-	3.5/2.07 eV	Rhodamine B	120 min	87.2%	[142]
BiFeO_3_/GdFeO_3_	-	2/2.3 eV	Methylene Blue	540 min	98%	[143]
CuO/BiFeO_3_	-	1.7/2.18 eV	Rhodamine B	270 min	50%	[144]
BiFeO_3_/ZnFe_2_O_4_	-	2.17/2.03 eV	Methylene Blue	120 min	97%	[145]
BiFeO_3_/TiO_2_	-	2.2/3.2 eV	Methylene Blue	180 min	94.4%	[146]
BiFeO_3_/Fe_2_O_3_	-	2.25/1.9 eV	Methylene Blue	60 min	94%	[147]
BiFeO_3_/CuWO_4_	-	2.1/2.3 eV	Methyl orange	120 min	85%	[148]
Ag_2_O/BiFeO_3_	-	1.3/2.1 eV	Rhodamine B	60 min	97%	[149]
BiFeO_3_/g-C_3_N_4_	-	2.13/2.78 eV	Rhodamine B	60 min	100%	[150]

It was reported that the co-substitution of Ce and Ni enormously impacts the photocatalytic efficiency of undoped BFO [134], which is maximized with the increase of co-doping levels. The best photocatalytic methylene blue and rhodamine B degradation efficiency were estimated at 93.29% and 96.05% after 90 min for Ce- and Ni-co-substitution BFO. The results suggest that the photocatalyst activity depends on the quantity of photon energy absorbed by the catalyst and the extent of the pollutant’s adsorption on the photocatalyst’s surface. The bandgap of Ce- and Ni-co-doped BFO is smaller than pristine bismuth ferrite, which helps absorb more energy than pure BFO. Meanwhile, the adsorption of pollutants on the photocatalyst surface is high for Ce–Ni-co-substitution BFO due to its larger porosity and raised surface area, which reveals a remarkable photocatalytic activity. The bandgap of undoped bismuth ferrite would be effectively reduced from 2.10 eV to 1.85 eV, which provides large photocatalytic efficiency under irradiation using various wavelengths of light. Depending on these experimental findings, the enhanced photocatalytic efficiency of Ce–Ni-co-substitution BFO would be ascribed to the raised optical absorption, the successful separation, and then the migration of photo-produced charge carriers with the reduced recombination feasibility of electron–hole pair findings from the co-substitution influence [134].

**Figure 4:**
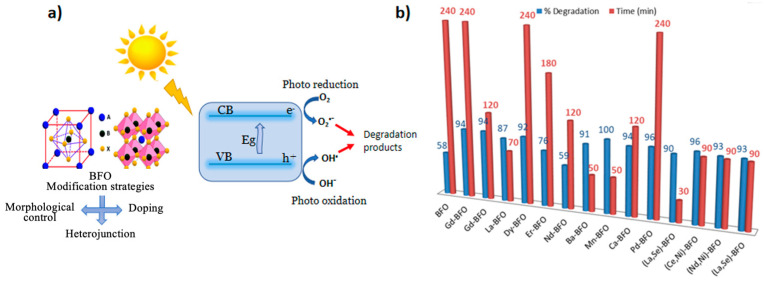
(**a**) Photocatalytic degradation of dye using doped BFO catalyst and (**b**) its degradation time and degradation efficiency.

Photocatalytic activities of (Nd, Ni)-co-doped BFO nanoparticles are determined through the degradation of methylene blue dye under visible light and H_2_O_2_ [135]. After 90 min reaction time, the degradation of MB is improved for (Nd, Ni)-co-doped BFO (93% degradation). A similar result was found for (Ba and Ca)-co-doped BFO [125]. Its photodegradation efficiency was found to be 93% after 90 min performed in the conditions of pH value 2 and with the addition of 0.5 mL H_2_O_2_. Basically, the efficiency of the photocatalysts depends on the nature of doping, which affects factors like the crystallite size, morphology, surface area, band gap (Figure 4a), and photo-induced electron–hole separation efficiency of the catalyst. Figure 4b summarizes the degradation time and efficiency of the BFO-doped elements reported in this review.

The crucial process of removing harmful pollutants from the environment is facilitated by the photodegradation of organic dyes. A vital role in this process is played by BFO-based heterostructures due to their unique properties, such as high photocatalytic activity and excellent stability. Visible light is effectively absorbed through these heterostructures and the electrons generated during the process react with the dye molecules, leading to their degradation. Furthermore, the synthesis of BFO-based heterostructures can be easily accomplished using simple methods, making them a cost-effective and sustainable solution for environmental remediation. The photodegradation of organic dyes using BFO-based heterojunctions has been studied extensively in recent years; various organic dyes have been subjected to this process, including methylene blue or orange, rhodamine B, and Congo red. Several factors should be considered when selecting an organic dye for photodegradation with a BFO heterostructure. These include the properties of the dye itself, such as its absorption spectrum and chemical stability, as well as the specific conditions of the photodegradation process, such as the light source and the presence of any co-catalysts. Ultimately, the choice of dye will depend on balancing these factors to achieve optimal performance and efficiency in the photodegradation process.

**Figure 5:**
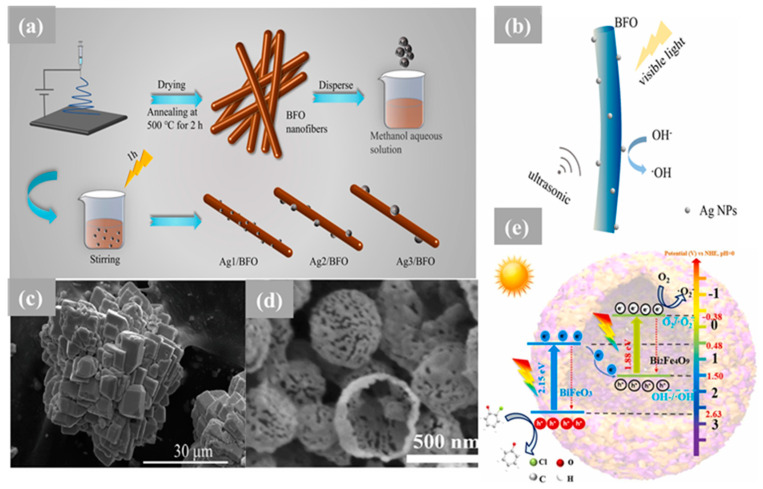
(**a**) Illustration of the synthesis procedure of the pure BFO nanofibers and Ag/BFO composites. (**b**) Schematic illustration of the synergy of plasmonic and piezotronic effects (reproduced with permission from Ref. [138]). (**c**) SEM image of BiFeO_3_-Ns (reproduced with permission from ref [146]). (**d**) SEM image of BiFeO_3_/Bi_2_Fe_4_O_9_ nanospheres. (**e**) S-scheme of the BiFeO_3_/Bi_2_Fe_4_O_9_ heterojunction hollow nanospheres with an Fe–O channel for enhancing charge separation to achieve high-efficiency photocatalytic o-chlorophenol degradation (reproduced with permission from Ref. [141]).

Nanocomposites of BiFeO_3_-GdFeO_3_ (BFO-GFO) heterostructures were synthesized for the first time utilizing the sol–gel technique and investigated for dye degradation [143]. According to Tauc plots, the band gap energies of BiFeO_3_-GdFeO_3_ were found to be 1.8 eV, while 2.0 eV and 2.3 eV were determined for BFO and GdFeO_3_, respectively [143]. The findings showed that when methylene blue was exposed to pure GdFeO_3_ for 9 h, its degradation was limited, indicating that GdFeO_3_ has a restricted photocatalytic activity under visible light. On the one hand, the decreased photodegradation efficiency of GdFeO_3_ was linked to its high band gap potential, inadequate absorption in the UV light range, and less-than-optimal photoelectric conversion. On the other hand, BFO was able to break down as much as 76% of the methylene blue after 9 h of irradiation. Remarkably, a high photodegradation efficiency of 98% for methylene blue was achieved for the BiFeO_3_-GdFeO_3_ composite after 9 h of irradiation. The improved photocatalytic performance of the BiFeO_3_-GdFeO_3_ composite could potentially be attributed to the formation of a heterojunction, hypothesized to induce the generation of photogenerated electron–hole pairs, resulting in an elevation in photocatalytic activity. Furthermore, the lowering of band gap values has been instrumental in enhancing its photodegradation efficiency by allowing improved visible-light absorption [143]. Xu et al. reported the rational design of Ag/BiFeO_3_ fibrous heterostructures using an electrospinning process, as illustrated in Figure 5a, with the aim of coupling piezoelectric and plasmonic effects (Figure 5b) to modulate the separation and migration of photogenerated charge carriers. It was demonstrated through PFM testing that the piezoelectric feature of 29.3 pm at −7.53 V was exhibited using the Ag_2_/BFO hybrid. Furthermore, when the ultrasound was introduced, the photocatalytic degradation rate of MO and MB over Ag_2_/BFO reached 96% and 95% within 100 min, respectively. The significant improvement in the photocatalytic activity was attributed to the synergistic effect of the piezoelectricity and LSPR, wherein the piezoelectric field within the BFO was found to further promote the directional migration and separation of photogenerated charge carriers induced through the LSPR effect of Ag NPs on the surface [138]. Through a combination of hydrothermal and post-impregnation techniques, the CuO/BFO composite with a p–n heterojunction structure was fabricated. In the context of the photocatalytic evaluation of methylorange degradation under visible light, it was observed that an optimal photocatalytic degradation efficiency of up to 50% was attained when the loading content of CuO was set at 15%, surpassing that of pure BFO and CuO by more than threefold. Furthermore, following five cycles of photodegradation of methyl orange, no significant loss of photocatalytic activity in CuO/BFO was observed, confirming its stability and long-term reusability [144]. In another piece of research, a BFO/MoS_2_ nanocomposite was successfully synthesized using a combination of the sol–gel procedure for BFO and the hydrothermal method for MoS_2_ [137]. The as-prepared BFO/MoS_2_ nanocomposite demonstrated a remarkable performance in the visible-light photo-decolorization of RhB. The photocatalytic experiments indicated that an impressive removal rate of approximately 89% of rhodamine B is achieved through the nanocomposite (50% BFO/50% MoS_2_ Wt) within 200 min. under visible-light irradiation. This exceptional photocatalytic activity can be ascribed to the highly efficient separation of photogenerated electron–hole pairs. Furthermore, the high activity is maintained by the BFO/MoS_2_ nanocomposite, even after undergoing three photoreaction cycles, and can be easily separated and collected using an external magnetic field [137]. In another study, a facile ultrasonic/hydrothermal route was employed to synthesize the BFO/BVO p–n junction, resulting in a significant improvement in the performance of n-type BVO and p-type BFO for the photocatalytic degradation of tetracycline (TC) and the photoelectrochemical (PEC) water splitting process [136]. Notably, the photodegradation of TC using BVO and BFO was found highly dependent on the pH level, while that using BFO/BVO exhibited pH-independent behavior. The introduction of BFO/BVO p–n junction nanostructures led to a significant improvement in TC photocatalytic degradation, achieving removal rates of 84% and 95% at pH 6.7 and 9.5, respectively, as compared with 31% and 22% with BFO alone. Moreover, an increase from 37% with BVO to 84% with the BFO/BVO p–n junction at pH  =  2.5 was demonstrated [136].

To enhance visible-light adsorption and photocatalytic activity, a modified BFO/rGO nanocomposite was fabricated via sol–gel process by controlling heat treatment parameters and rGO% [139]. When compared with BFO, BGO exhibits a narrower band gap energy of 1.8 eV, a lower rate of charge carrier recombination, and stronger magnetic characteristics. The highest photocatalytic activity at the optimum concentration was demonstrated by BGO with 1 wt% rGO in the range of photocatalysts prepared (1, 5, 10, and 20 wt% rGO), leading to MB degradation under visible light of up to 98% after 5 h [139]. Wang et al. reported a sonocatalytic removal of tetracycline using an S-scheme Cu_2_O/BFO heterojunction. BFO was synthesized through a simple solvothermal method, while Cu_2_O/BFO was fabricated through a co-precipitation method. The formation of heterojunctions between BFO and Cu_2_O was proved using photoluminescence (PL) spectroscopy, showing a low intensity in the case of BFO/Cu_2_O compared with pure BFO, which effectively inhibits the carrier recombination and improves the charge transfer efficiency. Superior sonocatalytic oxidation of TET is exhibited through CBF-3, with a degradation efficiency of TET reaching 98.0% under optimal conditions, such as a 1 g·L^−1^ of CBF-3 composite, a 20 mg·L^−1^ TET solution, a US irradiation power of 500 W, and a US irradiation time of 5 h [140].

To demonstrate the synergetic effect of the BFO ferroelectric property on the photocatalytic performance, a BiFeO_3_/TiO_2_ p-n heterojunction photocatalyst was developed through hydrolysis and precipitation. This approach resulted in the formation of TiO_2_ nanospheres on BFO nanocubes (Figure 5c) that contribute to enhancing the photocatalytic efficiency. Improved separation and transfer efficiency of photoelectron–hole pairs, higher sensitivity to visible light, and enlarged specific surface area are observed in the BiFeO_3_/TiO_2_ p-n heterojunction, as compared with neat TiO_2_ and BFO. Additionally, superior photocatalytic degradation performance for methylene blue (MB) and common antibiotic tetracycline (TC) under UV- and visible-light irradiation is exhibited through the composite. MB degradation rates of 78.4% and 90.4% under UV- and visible-light irradiation, respectively, are achieved within 3 h [146]. In another study, an effective approach to enhancing the charge separation for high-efficiency photocatalytic o-chlorophenol degradation is achieved through the fabrication of BiFeO_3_/Bi_2_Fe_4_O_9_ hollow nanosphere (Figure 5e) phase-mixed heterojunctions using a template-adsorption-calcination method. An S-scheme mechanism with an interesting Fe 3d-channel for efficient charge separation was confirmed to be followed by the BiFeO_3_/Bi_2_Fe_4_O_9_ heterojunction (Figure 5e). The resultant composite nanospheres were prepared through calcination in the air using a muffle furnace at temperatures ranging from 500 °C to 800 °C for a duration of 2 h. The as-prepared samples were designated as BFO-500, BFO-600, BFO-700, and BFO-800. Under visible-light irradiation, the optimized sample (designated BFO-700) showed 7.7- and 10.7-fold higher photoactivity than pure BiFeO_3_ and Bi_2_Fe_4_O_9_ nanoparticles, respectively. The enhanced photocatalytic activity of BFO-700 can be attributed to several factors, including increased light absorption due to the hollow structure, enhanced charge separation facilitated by the S-scheme mechanism using Fe–O channels, and preferential dechlorination through selective adsorption [141].

### 3.2. Solar Water Splitting

The photocatalytic water splitting process is the conversion of solar energy into chemical energy used to drive the production of H_2_ and O_2_. This process is achieved when the photocatalyst absorbs natural solar light (i.e., sunlight) while dispersed in water and then electron–hole pairs migrate to the surface of the photocatalyst to generate and produce H_2_ and O_2_ [151]. The photocatalytic dissociation of water has many advantages, such as being suited to splitting water of a nearly neutral pH in a one-step process without the need for an applied external bias. However, unassisted overall water splitting under a single-absorber photocatalytic process must achieve the following two conditions: (1) the valence and conduction gap edges of this photocatalyst must astride across the water oxidation (redox) and proton reduction and potentials; (2) this photocatalyst must possess an adequate narrow bandgap to absorb a majority of the solar spectrum [59]. Considering the potential of water splitting, the lowest energy of the absorbed photon must be larger than 1.23 eV to trigger this reaction. In view of the energy requirements set by H_2_O reduction and oxidation potentials of the conduction band and valence band levels, the optimal band gap of the semiconductor for efficient H_2_ production is about 2.0 eV [120]. Therefore, developing a new photocatalytic material with an adequate band gap that can directly split water into H_2_ under visible-light irradiation is essential for H_2_ production. BFO is an interesting multiferroic material for energy-related applications, especially H_2_ generation, through photocatalytic water splitting due to its small band gap (~2.2 eV) [152], good carrier transport properties, and large absorption of visible light extending up to 750 nm.

Through the systemic investigation of the Sr-doping level of BFO, it is found that the HER enhancement originates from the improvement of ferromagnetism of Sr-doped BFO without the obvious scarification of ferroelectricity at room temperature [152]. The H_2_ evolution of Sm-5%-doped BFO has also been elucidated recently [153]. The rate of H_2_ production has been found to be 6.54 μmol·h^−1^·cm^−2^. The improved photocatalytic activity of Sm-5%-doped BFO has been explained based on the effect of doping, better solar spectral response, hindering the recombination loss of photo-generated charge carriers, and fast and facile charge transport.

**Figure 6:**
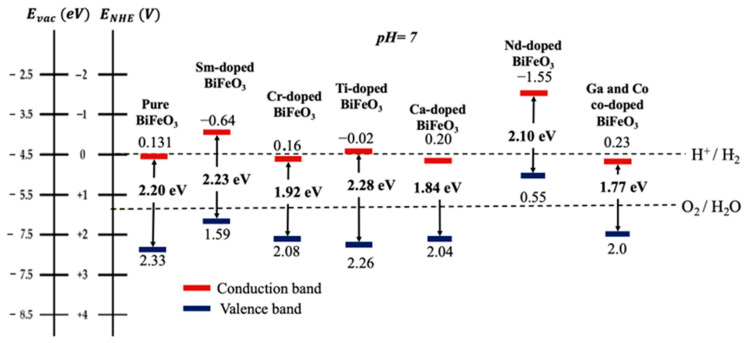
Band gap positions with respect to valence and conduction band at pH = 7 of pure BFO, Sm-doped BFO, Cr-doped BFO, Ti-doped BFO, Ca-doped BFO, Nd-doped BFO, Ga- and Co-co-doped BFO.

To enhance the photocatalytic dissociation of water splitting, a new perovskite material has been reported by doping Gd in place of Bi and Co in place of Fe for H_2_ production through the photoelectrochemical splitting of water [154]. The doping levels lead to the band gap engineering from 2.23 eV to 1.77 eV, as shown in Figure 6. This band gap lowering improves the photocatalytic response of the resulting materials. The highest H_2_ production rate of 74.57 mmol·h^−1^·cm^−2^ has been found for Gd- and Co-co-doped BFO possessing the lowest band gap of 1.77 eV, with a maximum photo-conversion efficiency of 2.29%. Thus, the higher rate of H_2_ production and better photo-conversion efficiency of Co-co-doped BFO is due to its better solar spectral response.

**Figure 7:**
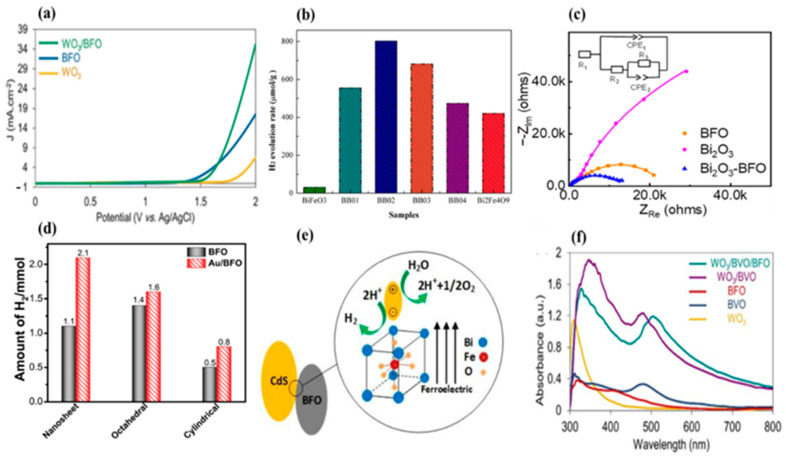
(**a**) LSV curves of WO_3_, BFO, and WO_3_/BFO photoanodes (reproduced with permission from Ref. [155]). (**b**) Stable hydrogen evolution from water using BiFeO_3_, Bi_2_Fe_4_O_9_, and BiFeO_3/_Bi_2_Fe_4_O_9_ heterojunction nanofibers under visible-light irradiation (λ > 420 nm) (reprinted with permission from Ref. [157]). (**c**) Photoelectrochemical impedance spectra (PEIS) of BFO, Bi_2_O_3_, and Bi_2_O_3_/BFO films (reprinted with permission from Ref. [158]). (**d**) Comparative H_2_ generation data after 2 h visible-light irradiation using the catalysts of BFO nanosheets (BFO-Ns), a BFO octahedron (BFO-Oct), a cylindrical-shaped BFO (BFO-Cyl), and their heterostructures as Au/BFO-Ns, Au/BFO-Oct, and Au/BFO-Cyl (reprinted with permission from Ref. [159]). (**e**) Effect of internal electrical field of the BFO on charge separation in CdS (reprinted with permission from Ref. [160]). (**f**) UV–vis absorption spectra of WO_3_, BVO, BFO, WO_3_/BVO, and WO_3_/BVO/BFO photoanodes (reprinted with permission from Ref. [161]).

The construction of heterojunctions is deemed a prospective methodology for the development of innovative photocatalysts for solar water splitting with outstanding performance. It involves combining different semiconductor materials with unequal band structures and suitable band alignment to form a junction interface that can promote the separation of photogenerated electron–hole pairs, leading to enhanced photocatalytic activity. WO_3_/BiFeO_3_ n-p heterojunction films were prepared using the sol–gel spin coating method [155]. Using 2-methoxyethanol as a solvent and DEA as an additive, the best BFO phase has been obtained with regard to impurity phases, micro-structural morphology, and photocurrents. The photocurrent exhibited through the WO_3_/BFO n-p heterojunction (35.2 mA⋅cm^−2^) shows a significant improvement over the photocurrents of neat WO_3_ (6.5 mA⋅cm^−2^) and BFO (17.5 mA⋅cm^−2^) thin films (Figure 7a) [155]. In another study, a facile ultrasonic/hydrothermal route was employed to synthesize the BFO/BVO p–n junction, resulting in a significant improvement in the performance of n-type BVO and p-type BFO for the photoelectrochemical (PEC) water splitting process [136]. The BFO/BVO nanostructures exhibited a favorable photocurrent density of 0.36 mA⋅cm^−2^ under UV–vis light and 0.23 mA⋅cm^−2^ under visible light at 1.0 V vs. Ag/AgCl [136]. In addition, a simple sol–gel process was used to synthesize a single-phase BFO film on a TiO_2_ photoanode to enhance photoelectrochemical (PEC) water splitting efficiency. The controllable thickness of the BFO films facilitated the induction of a significant ferroelectric polarization under bias voltage, thereby effectively adjusting the electric band bending at the BFO/TiO_2_ interface. As a result of this approach, the photocurrent density achieved using the BFO-5/TiO_2_ photoanode reached an impressive value of 11.25 mA⋅cm^−2^, surpassing that of bare TiO_2_ by over 20-fold. Furthermore, when the BFO-5/TiO_2_ photoanode was positively poled, it demonstrated a remarkable photocurrent density of 28.75 mA⋅cm^−2^ at 1.5 V vs. SCE under AM 1.5G illumination [156].

Zhang et al. fabricated BiFeO_3_/Bi_2_Fe_4_O_9_ heterojunction nanofibers through a facile wet chemical process followed by an electro-spinning technique. The inclusion of Bi_2_Fe_4_O_9_ within the BFO matrix resulted in a red-shift of its absorption edge, thereby enabling the enhanced absorption of visible light and improved efficiency in the separation of photogenerated carriers. Furthermore, the synthesized BiFeO_3_/Bi_2_Fe_4_O_9_ nanofibers exhibited heightened photocatalytic activity in the generation of H_2_ from water under visible-light irradiation conditions. Notably, the BiFeO_3_/Bi_2_Fe_4_O_9_ (BB02) sample demonstrated H_2_ evolution rates (~800 μmol·g^−1^) approximately 2.7 times and 2.0 times higher than those observed for pure BiFeO_3_ and pure Bi_2_Fe_4_O_9_ samples, respectively (Figure 7b). It was observed that the photocurrent density of the BB02 sample reached 1.8 μA⋅cm^−2^, far exceeding those achieved through pure BiFeO_3_ (0.6 μA⋅cm^−2^) and pure Bi_2_Fe_4_O_9_ (0.9 μA⋅cm^−2^) samples, respectively. Notably, the order of variation in photocurrent density was identified as follows: BB02 > BB03 > BB01 > BB04 > Bi_2_Fe_4_O_9_ > BFO [157]. More recently, high-quality Bi_2_O_3_, BFO, Bi_2_O_3_/BFO films on indium tin oxide (ITO) were produced using pulse laser deposition (PLD). It was observed that the BFO film exhibited both cathodic and anodic photocurrents in the potential range of −0.7–0.2 V compared with the Ag/AgCl reference electrode. Notably, the photocathodic current was significantly higher, indicating pronounced p-type photocathodic behavior suitable for potential photoelectrochemical (PEC) applications. In particular, the BFO film exhibited an onset potential of around −0.10 V versus the Ag/AgCl reference electrode, with a photocurrent density of −40.1 μA⋅cm^−2^ obtained at −0.68 V versus the Ag/AgCl reference electrode. Remarkably, the cathodic photocurrent density showed a significant increase in the Bi_2_O_3_/BFO heterojunction film compared with the BFO film, reaching a value as high as −84.07 μA⋅cm^−2^ at −0.68 V compared with the Ag/AgCl reference electrode. This clear improvement represents a doubling of the corresponding value obtained for the BFO film at the same potential, with an onset potential of 0.14 V relative to the Ag/AgCl reference electrode. In addition, the effect of Bi_2_O_3_ overlayer thickness was explored, showing that the maximum photocurrent is achieved for 4 nm Bi_2_O_3_/BFO. Moreover, electrochemical impedance spectroscopy (EIS) results illustrated interfacial charge transfer processes on the photoelectrodes. The equivalent circuit utilized in the analysis comprises an electrolyte resistor (R_1_), a ground resistor (R_2_), a charge transfer resistor (R_3_), and two constant-phase elements (CPE_1_ and CPE_2_). As shown in Figure 7c, the findings indicate a significant reduction in the low-frequency arc when transitioning from Bi_2_O_3_ to BFO and Bi_2_O_3_/BFO heterojunction films. The charge transfer resistance, denoted as R_3_, in the Bi_2_O_3_/BFO film measures approximately 10 kΩ, a value lower than the approximate 18 kΩ observed in the BFO film. Thus, a more rapid charge separation process can be achieved by adding a Bi_2_O_3_ overlayer onto BFO film [158].

Zhu et al. reported a method for the construction of a BiFeO_3_/Cu_2_O heterojunction on BFO matrix through the sol–gel route and magnetron sputtering. They showed an enhanced photoelectrochemical performance of BFO due to the effective separation of photogenerated electron–hole pairs. Under an applied voltage of −0.4 V vs. Ag/AgCl, the photocurrent density of BFO increased from −15 μA⋅cm^−2^ to −103 μA⋅cm^−2^. Furthermore, upon positive poling, the photocurrent density experienced a further escalation to exceed 200 μA⋅cm^−2^ [162]. To improve the photocatalytic efficiency, Au/BFO heterostructures were synthesized with different shapes through a simple hydrothermal method followed by radiolysis without the use of a surfactant or strong reducing agent. In the investigation of shape-dependent photocatalysis, it was revealed that higher H_2_ generation (1.4 mmol·h^−1^·g^−1^) was achieved using the BFO octahedron (BFO-Oct) in comparison with the H_2_ generation rates observed for the BFO nanosheets (BFO-Ns) (1.1 mmol·h^−1^·g^−1^) and the BFO cylindrical-shaped (BFO-Cyl) nanostructures (0.5 mmol·h^−1^·g^−1^) (Figure 7d). Following that, the effect of pH on the photocatalytic H_2_ evolution was examined to better understand the reaction conditions. An enhanced catalytic activity for H_2_ evolution was observed at pH 3, with approximately 2.4 mmol of H_2_ produced. This effect was attributed to the increased availability of H^+^ ions in the acidic solution. Conversely, as the medium shifted towards alkalinity, a decrease in H_2_ evolution activity was observed, primarily resulting from the insufficient presence of protons. Subsequently, following the radiolytic construction of the Au/BFO heterostructure, a notable enhancement in H_2_ generation efficiency was achieved, primarily as a consequence of photoinduced electron transfer due to surface-plasmon effect, which is a collective oscillation of conduction band electrons brought on by the interaction with visible-light photons. This phenomenon creates a local electromagnetic field at the BFO photocatalyst interface and subsequently increases the efficiency of charge-carrier separation [163]. When plasmonic metal nanoparticles are introduced to a certain distance, their localized electromagnetic field may couple and produce a significant number of hot electrons that significantly enhance photocatalytic reactions [164]. Additionally, the photo-response of the Au/BFO heterostructures was evaluated through photoelectrochemical (PEC) measurements in terms of the photocurrent. The results indicated that Au/BFO-Ns yielded higher photocurrents than BFO nanostructures, with an increase that was approximately 3.8-fold. In the absence of light, the current density for BFO-Ns remains negligible at 0.07 μA⋅cm^−2^. However, under continuous light illumination, the current density increases to 0.56 μA⋅cm^−2^ for BFO-Ns and 2.14 μA⋅cm^−2^ for Au/BFO-Ns at a potential of 0.6 V vs. Ag/AgCl, resulting in a remarkable 3.8-fold enhancement for Au/BFO-Ns heterostructures. In a similar study, Au/BFO-Ns heterostructures showed a considerably higher current density of approximately 235 μA⋅cm^−2^, indicating the generation of H_2_ through water reduction. In contrast, bare Au NPs exhibited a relatively lower current density of 75 μA⋅cm^−2^ at negative potential. Hence, the presence of Au NPs and their strong interaction with BFO-Ns likely contributed to the substantial current density observed in the Au/BFO-Ns heterostructures [159]. In a separate investigation, the photocatalytic performance of CdS for water splitting was improved by coupling with BFO, which led to the creation of a direct Z-scheme heterojunction [160]. The photocarrier transfer pathway entails the migration of CB electrons from CdS, characterized by a lower CB potential, to the VB of BFO, which exhibits a higher VB potential. Subsequently, these transferred electrons engage in recombination with holes, thereby generating charge carriers possessing elevated redox potentials within BFO. The accumulation of photoinduced electrons in the CB of CdS establishes an electron-rich zone, markedly mitigating CdS’s susceptibility to photo-oxidation. Conversely, the gathering of photoinduced holes in the VB of BFO creates a hole-rich region, affording protection to BFO against photo-reduction. Consequently, BFO is expected to demonstrate robust resistance to photo-oxidation, while CdS should exhibit substantial resistance to photo-reduction. Furthermore, as a result of the p-type conductivity of BFO and the n-type conductivity of CdS, an internal field formed in its crystal structure (Figure 7e) that contributed to lowering the hole recombination. Using hydrothermal and precipitation methods, BiFeO_3_ (BFO) and Cadmium Sulfide (CdS) were synthesized, respectively. A H_2_ evolution rate of 99.3 μmol·h^−1^·g^−1^ was observed with pure CdS. A low activity level was observed with pure BFO. This is due to the fact that BFO’s CB is not sufficiently negative to effectively reduce H^+^ into H_2_. When 30% BFO was added to CdS (CB-70), an increase in the H_2_ evolution efficiency to 263.2 μmol·h^−1^·g^−1^ was achieved. Further addition of BFO to reach 50% resulted in the evolution of 600 μmol·h^−1^·g^−1^ of H_2_, which represents the optimal outcome [160]. Table 5 summarizes the different BFO-based materials tested for solar water splitting.

Recent research has demonstrated that it is possible to design a complex system comprising more than two semiconductors. In this context, a triple heterojunction photoanode comprising WO_3_/BiVO_4_ (BVO)/BiFeO_3_ (BFO) porous layers was fabricated and deposited on an FTO glass substrate using a sol–gel spin-coating technique. The band gap energy values for pure WO_3_, BVO, and BFO of 3, 2.42, and 2.14 eV, respectively, were measured. The WO_3_/BVO/BFO heterojunction photoanode presents much higher solar water splitting performance with a maximum photocurrent of 46.9 mA⋅cm^−2^ at 2.53 V vs. RHE, in contrast to that of the individual component following the order BFO > WO_3_ > BVO with their corresponding photocurrent values of 17.5 > 6.5 > 4.6 mA⋅cm^−2^ at the same potential. Nevertheless, the photocurrent of the WO_3_/BVO double-layer sample was slightly higher than that of the WO_3_/BVO/BFO sample at 1.23 V vs. RHE, possibly as a result of the reduced porosity caused by BFO deposition. This reduction in surface area subsequently impacts the interaction between the electrolyte and the photoanode. Furthermore, due to scattering and light trapping, the light absorbance of the double layer heterojunction is greater than that of the triple layer heterojunction (Figure 7f). In fact, the WO_3_/BVO/BFO triple layer shows a much higher photocurrent at higher voltages, which can be attributed to the formation of a p-n junction and a self-biasing field of the BFO [161]. To boost the photocatalytic performance, Xu et al. developed a Z-scheme core@shell heterostructure piezo-photocatalyst for the first time by combining covalent organic frameworks (COFs) and piezoelectric material which is BFO-based. The BiFeO_3_@TpPa-1-COF (BFO@COF20 C) photocatalyst demonstrated exceptional H_2_ and O_2_ generation rates of 1416.4 and 708.2 mol·h^–1^·g^–1^, respectively, under ultrasonication and simulated solar irradiation, exceeding the previously reported photocatalysts and piezoelectric materials for solar water splitting [168].

## 4. Summary and Outlook

This work demonstrated the outstanding physico-chemical properties of BFO-based nanomaterials, in particular their unique electronic structure and crystal symmetry. These characteristics play a major role in their remarkable multiferroic properties at room temperature, making them standout materials exhibiting high photocatalytic performances in the entire visible region of the solar spectrum. In the pursuit of optimizing their photocatalytic performances, alloying, substitution, and doping are among the strategies adopted to adjust the energy band structure leading to an improved photocatalytic performance. Based on our investigations, doping appeared to be more effective for tailoring their photocatalytic properties for enhanced dye photodegradation, while forming heterojunctions is more suitable for solar-driven water splitting.

Yet, these multiferroic nanomaterials were not evaluated through an operando condition such as under external electric field excitation to take advantage of the high polarization they have that would yield a further enhancement in their photocatalytic performance. We believe that future developments need to take this aspect into account and propose novel experimental setups that include electrical and/or magnetic field monitoring during the photocatalysis process. It has to be noted that this class of materials belong to the transition metal oxide perovskites that have tunable physical properties while being very stable under aggressive environment making them the future electrodes for high-performing photoelectrocatalytic reactions.

## Figures and Tables

**Table 1:** Effect of BFO grains and particle size on its dielectric constant and remanent polarization.

Material	Route	Precursors	Size (nm)	ԑ_r_ RT; 10^2^ Hz	P_r_ (µC·cm^−2^)	Ref.
BiFeO_3_ bulk	Solid-state	Bi_2_O_3_, Fe_2_O_3_	-	-	40	[80]
BiFeO_3_ single cristal	Spontaneous crystallization	Bi_2_O_3_, Fe_2_O_3_	-	-	75	[81]
BiFeO_3_	Solid-state	Bi_2_O_3_, Fe_2_O_3_	10^3^	-	8.9	[82]
BiFeO_3_	Solid-state	Bi_2_O_3_, Fe_2_O_3_	2 × 10^2^	25	7.5	[83]
BiFeO_3_	Combustion	Bi(NO_3_)_3_·5H_2_O, Fe(NO_3_)_3_·9H_2_O, Organic fuel	40	1118	-	[84]
BiFeO_3_	Sol-gel	Bi(NO_3_)_3_, 5H_2_O/Fe(NO_3_)_3_, 9H_2_O	12	84.5	8.2	[85]
BiFeO_3_	Solvothermal	BiCl_3_, FeCl_3_·6H_2_O, HMTA	-	4000	6.7	[86]
BiFeO_3_ single cristal	Solid-state	Bi_2_O_3_, Fe_2_O_3_	10^6^	-	6.1	[87]
BiFeO_3_	Solid-state	Bi_2_O_3_, Fe_2_O_3_	2 × 10^3^	121	0.3	[88]
BiFeO_3_	Combustion	Bi(NO_3_)_3_, 5H_2_O/Fe(NO_3_)_3_, 9H_2_O	47	91	-	[89]

**Table 2:** Effect of BFO grains and particle size on its dielectric constant and remanent polarization.

Material	Route	Precursors	Size (nm)	ԑ_r_ RT; 10^2^ Hz	P_r_ (µC·cm^−2^)	Ref.
Bi_0.8_La_0.2_FeO_3_	Hydrothermal	Bi(NO_3_)_3_, Fe(NO_3_)_3_, La(NO_3_)_3_, KOH	10^5^	225	-	[90]
Bi_0.95_La_0.05_FeO_3_	Sol–gel	Bi(NO_3_)_3_·5H_2_O, Fe(NO_3_)_3_·9H_2_O, La(NO_3_)_3_⋅6H_2_O	4 × 10^2^	50,000	-	[91]
Bi_0.98_La_0.02_FeO_3_	Sol–gel	Bi(NO_3_)_3_·5H_2_O, Fe(NO_3_)_3_·9H_2_O, La(NO_3_)_3_⋅6H_2_O	80	161.8	140	[92]
Bi_0.8_Y_0.2_FeO_3_	Sol–gel & combustion	Bi(NO_3_)_3_·5H_2_O, Fe(NO_3_)_3_·9H_2_O, Y(NO_3_)_3_⋅6H_2_O	41	500	16	[93]
Bi_0.85_Gd_0.15_FeO_3_	Sol–gel	Bi(NO_3_)_3_·5H_2_O, Fe(NO_3_)_3_·9H_2_O, Gd(NO_3_)_3_.3H_2_O	16	2193	7	[94]
Bi_0.97_Yb_0.03_FeO_3_& Bi_0.9_Yb_0.1_FeO_3_	Hydrothermal	Bi(NO_3_)_3_, Fe(NO_3_)_3_, Yb(NO_3_)_3_, KOH	29	150	0.4	[95]
Bi_0.985_Ba_0.015_FeO_3_	Hydrothermal	Bi(NO_3_)_3_, Fe(NO_3_)_3_, Ba(NO₃)₂, KOH	57	125	-	[96]
Bi_0.97_Pb_0.03_FeO_3_	Precipitation	Bi(NO_3_)_3_·5H_2_O, Fe(NO_3_)_3_·9H_2_O, Pb(NO_3_)_3_·5H_2_O,	-	2500	0.8	[97]
Bi_0.9_Eu_0.1_FeO_3_	Microwave-assisted	Bi(NO_3_)_3_·5H_2_O, Fe(NO_3_)_3_·9H_2_O, Eu(NO_3_)_3_⋅6H_2_O	18	150	-	[98]
BiFe_0.95_Ti_0.05_O_3_	Solvothermal	Bi(NO_3_)_3_·5H_2_O, Fe(NO_3_)_3_·9H_2_O, TiO_2_	7 × 10^2^	1000	-	[100]
BiFe_0.85_ Hf(_3/4_)_0.15_O_3_	Combustion	Bi(NO_3_)_3_·5H_2_O, Fe(NO_3_)_3_·9H_2_O, HfCl_4_	30	-	0.2	[106]
BiFe_0.975_Zr_0.025_O_3_	Hydrothermal	Bi(NO_3_)_3_·5H_2_O, Fe(NO_3_)_3_·9H_2_O, ZrOCl_2_.8H_2_O	46	366	-	[101]
BiFe_0.99_Ni_0.01_O_3_	Sol–gel	Bi(NO_3_)_3_·5H_2_O, Fe(NO_3_)_3_·9H_2_O, Ni(NO_3_)_3_⋅6H_2_O	-	2000	2.6	[102]
Bi_0.99_La_0.1_Fe_0.95_Ni_0.05_O_3_	Solid-state	Bi_2_O_3_, Fe_2_O_3_, La_2_O_3_, & NiO	-	2083	0.2	[103]
Bi_0.99_Ba_0.1_Fe_0.99_Nb_0.1_O_3_	Sol–gel & combustion	Bi(NO_3_)_3_, Fe(NO_3_)_3_, Ba(NO₃)₂, C_6_H_4_NNbO_12_	27	115	3.2	[104]
Bi_0.9_La_0.075_Ce_0.025_FeO_3_	Combustion	Bi(NO_3_)_3_·5H_2_O, Fe(NO_3_)_3_·9H_2_O, La(NO_3_)_3_⋅6H_2_O, Ce(NO_3_)_3_⋅6H_2_O	25	105	3.2	[107]
Bi_0.85_Er_0.15_FeO_3_	Solid-state	Bi_2_O_3_, Fe_2_O_3_, Er_2_O_3_,	-	500	0.1	[99]
BiFe_0.99_(Li_0⋅5_Nb_0.5_)_0.01_O_3_	Ceramic sintering	Bi_2_O_3_, Fe_2_O_3,_ Li_2_O, Nb_2_O_5_	-	1050	0.2	[105]

**Table 3:** BFO-based heterostructures properties, dielectric constant (ԑr), and remanent polarization (Pr).

Material	Route	Precursors	Size (nm)	ԑ_r_ RT; 10^2^ Hz	P_r_ (µC·cm^−2^)	Ref.
(BiFeO_3_)_0.6_(CaTiO_3_)_0.4_	Solid-state	CaCO_3_, Bi_2_O_3_, TiO_2_, Fe_2_O_3_	-	1075	0.1	[108]
(Bi_0.95_Nd_0.05_FeO_3_)_0.8_(PbTiO_3_)_0.2_	Solid-state	Bi_2_O_3_, Nd_2_O_3_, Fe_2_O_3_, PbO, TiO_2_	2 × 10^2^	1625	0.8	[109]
Mn-doped-(BiFeO_3_)_0.5_(SrTiO_3_)_0.5_	Solid-state	Bi_2_O_3_, Fe_2_O_3_, SrO, TiO_2_, MnO_2_	-	720	6	[110]
(BiFeO_3_)_0.65_(BaTiO_3_)_0.35_	Solid-state	BaCO_3_, TiO_2_, Bi_2_O_3_, Fe_2_O_3_	3 × 10^2^	4300	3.7	[111]
(BiFeO_3_)_0.66_(PbTiO_3_)_0.34_	Sol–gel	Bi(NO_3_)_3_·5H_2_O, Fe(NO_3_)_3_·9H_2_O, Pb(CH_3_COO)_2_·3H_2_O, Ti[OCH(CH_3_)_2_]_4_	-	587	95	[112]
[(Bi_0.9_Dy_0.1_)FeO_3_]_0.5_–(PbTiO_3_)_0.5_	Combustion	PbO, Bi_2_O_3_, Fe_2_O_3_, Dy_2_O_3_, TiO_2_	-	103	9	[115]
(BiFeO_3_)_0.8_–(GdMnO_3_)_0.2_	Combustion	Fe(NO_3_)_3_·9H_2_O, Bi(NO_3_)_3_.5H_2_O, Gd_2_O_3_, (CH_3_COO)_2_ Mn·4H_2_O	25	688	0.4	[116]
(BiFeO_3_)_0.7_-PbTiO_3_)_0.3_	Solid-state	Bi_2_O_3_, Fe_2_O_3_, PbO, TiO_2_, SrCO_3_	10^3^	295	7	[117]
(Na_0.5_Bi_0.5_TiO_3_)_0.775_–(SrTiO_3_)_0.2_–BiFeO_3_)_0.025_	Solid-state	Na_2_CO_3_, Bi_2_O_3_, SrCO_3_, Fe_2_O_3_, TiO_2_	-	-	24	[118]
0.655BiFeO_3_–0.025BiCoO_3_–0.32BaTiO_3_	Solid-state	Bi_2_O_3_, Fe_2_O_3_, Co_3_O_4_, La_2_O_3_, BaCO_3_, TiO_2_	60	2000	5	[113]
0.675BiFeO_3_–0.3BaTiO_3_–0.025LaFeO_3_–1.25Ta_2_O_5_	Solid-state	B_i2_O_3_, Fe_2_O_3_, La_2_O_3_, TiO_2_, Ta_2_O_5_, BaCO_3_	-	1149	2	[114]

**Table 5:** BFO-based materials for solar water splitting.

Material	Application	Band Gap (ev)	HER/ Efficiency	Photocurrent Density	Refs.
BiFeO_3_	Solar WS	2.2	−	40 μA⋅cm^−2^ @ 0.6 V	[72]
Bi_0.95_Sm_0.05_FeO_3_	Photoelectrocatalytic	2.2	−	0.11 mA⋅cm^−2^	[153]
BiFe_0.9_Cr_0.1_O_3_	Solar cells	1.9	−	0.3 mA⋅cm^−2^	[165]
BiFe_0.9_Ti_0.1_O_3_	Solar WS	2.3	−	−	[166]
Bi_0.85_Sr_0.15_FeO_3_	Solar WS	−	−	0.5 mA⋅cm^−2^ @ 1.4 V	[152]
Bi_0.97_Y_0.03_FeO_3_	Solar WS	−	−	0.7 mA⋅cm^−2^ @ 1.4V	[167]
Bi_0.875_SrxFe_0.875_Ti_0.125_O_3_	Solar WS	2.5	191 μmol·h^−1^·g^−1^/ −	0.2 μA⋅cm^−2^	[76]
Bi_0.75_Gd_0.25_Fe_1−y_Co_y_O_3_	Solar WS	1.8	74.6 μmol·h^−1^·cm^−2^/ −	2 mA⋅cm^−2^ @ 1 V	[154]
WO_3_/BiFeO_3_	Solar WS	3/2.2	−	35.2 mA⋅cm^−2^ @ 2 V	[155]
BiFeO_3_/BiVO_4_	Solar WS	2.7	−	0.2 mA⋅cm^−2^ @ 1 V	[136]
Bi_2_O_3_/BiFeO_3_	Solar WS	2.8/2.7	−	−84 μA⋅cm^−2^ @ −0.7 V	[158]
BiFeO_3_/Cu_2_O	Solar WS	2.6	−	−0.5 mA⋅cm^−2^ @−0.7 V	[162]
BiFeO_3_/TiO_2_	Solar WS	2.1/3.2	−	28.8 mA⋅cm^−2^ @ 1.5 V	[156]
BiFeO_3_/Bi_2_Fe_4_O_9_	Solar WS	2.2/1.9	800 μmol·g^−1^ for 8h/ −	1.8 μA⋅cm^−2^	[157]
Au/BiFeO_3_	Solar WS	2.1	2.1 mmol·h^−1^ for 2h/ −	2.1 μA⋅cm^−2^ @ 0.6 V	[159]
BiFeO_3_@COF Z-Scheme	Solar WS	−	1416.4 μmol·h^−1^·g^−1/^ −	3.8 μA⋅cm^−2^ @ 0.6 V	[168]
WO_3_/BiVO_4_/BiFeO_3_	Solar WS	3/2.4/2.1	−	47 mA⋅cm^−2^ @ 2.5 V	[161]
CdS/BiFeO_3_	Solar WS	2.4/2.1	600 μmol·h^−1^·g^−1/^ −	1.2 mA⋅cm^−2^	[160]
g-C_3_N_4_/BiFeO_3_ Z-scheme	Solar WS	2.8/2.3	23.31 μmol·h^−1^·g^−1^/ −	−	[169]
Z-scheme Au-LaFeO_3_-g-C_3_N_4_-BiFeO_3_	Solar WS	2/2.7/2	698.4 μmol·h^−1^·g^−1/^ −	1.2 μA⋅cm^−2^	[170]
BiFeO_3_/ZnIn_2_S_4_ Z-scheme	Solar WS	2/2.3	87.3 μmol·h^−1^·g^−1/^ −	0.5 μA⋅cm^−2^	[171]

## Data Availability

Data are available upon request to the corresponding author.

## Abbreviations

AM 1.5G	Air Mass 1.5 Global Spectrum
CB	Conduction Band
CPE	Constant Phase Elements
CS	Conventional Sintering
DEA	Diethaloamine
EIS	Electrochemical Impedance Spectroscopy
FTO	Fluorine doped Tin Oxide
HER	Hydrogen Evolution Reaction
ITO	Indium Tin Oxide
LSV	Linear Sweep Voltammetry
MB	Methylene Blue
MWS	Microwave Sintering
NPs	Nanoparticles
OER	Oxygen Evolution Reaction
PC	Photocatalysis
PEC	Photoelectrochemical
pH	Potential of Hydrogen
PL	Photoluminescence
PLD	Pulsed Laser Deposition
PMs	Perovskite materials
P_r_	Remanent Polarization
PV	Photovoltaic
RHE	Reversible Hydrogen Electrode
ROS	Reactive Oxygen Species
RT	Room Temperature
SCE	Saturated Calomel Electrode
SEM	Scanning Electron Microscope
SG	Sol–gel
STH	Solar-to-Hydrogen
TC	Tetracycline hydrochloride
UV	Ultra Violet
VB	Valence Band
WS	Water Splitting
ε_r_	Dielectric Constant

## References

[1] Barbir F., Veziro T.N., Plass H.J. (1990). Environmental damage due to fossil fuels use. Int. J. Hydrogen Energy.

[2] Hassan A., Ilyas S.Z., Jalil A., Ullah Z. (2021). Monetization of the environmental damage caused by fossil fuels. Environ. Sci. Pollut. Res..

[3] Geerken T.G., Timmermans V.T., Lassaux S.L. (2005). Hydrogen and its Applications: Review of Life Cycle Assessment Studies and Well-to-Wheel Studies. Hysociety.

[4] Burdack A., Duarte-Herrera L., López-Jiménez G., Polklas T., Vasco-Echeverri O. (2022). Techno-economic calculation of green hydrogen production and export from Colombia. Int. J. Hydrogen Energy.

[5] Shahin M.S., Orhan M.F., Saka K., Hamada A.T., Uygul F. (2023). Energy assessment of an integrated hydrogen production system. Int. J. Thermofluids.

[6] Younas M., Shafique S., Faisal A., Hafeez A., Javed F., Mustafa M., Rehman F. (2023). Hydrogen Production through Water Vapors using Optimized Corona-DBD Hybrid Plasma Micro-Reactor. Fuel.

[7] Hren R., Vujanović A., Van Fan Y., Klemeš J.J., Krajnc D., Čuček L. (2023). Hydrogen production, storage and transport for renewable energy and chemicals: An environmental footprint assessment. Renew. Sustain. Energy Rev..

[8] Martins F., Felgueiras C., Smitkova M., Caetano N. (2019). Analysis of Fossil Fuel Energy Consumption and Environmental Impacts in European Countries. Energies.

[9] Younas M., Shafique S., Hafeez A., Javed F., Rehman F. (2022). An Overview of Hydrogen Production: Current Status, Potential, and Challenges. Fuel.

[10] Ardo F.M., Lim J.W., Ramli A., Lam M.K., Kiatkittipong W., Abdelfattah E.A., Shahid M.K., Usman A., Wongsakulphasatch S., Sahrin N.T. (2022). A review in redressing challenges to produce sustainable hydrogen from microalgae for aviation industry. Fuel.

[11] Aydin M.I., Dincer I. (2022). An assessment study on various clean hydrogen production methods. Energy.

[12] Mehanovic D., Peloquin J.F., Dufault J.F., Fréchette L., Picard M. (2022). Comparative techno-economic study of typically combustion-less hydrogen production alternatives. Int. J. Hydrogen Energy.

[13] Midilli A., Kucuk H., Topal M.E., Akbulut U., Dincer I. (2021). A comprehensive review on hydrogen production from coal gasification: Challenges and Opportunities. Int. J. Hydrogen Energy.

[14] Dincer I., Acar C. (2014). Review and evaluation of hydrogen production methods for better sustainability. Int. J. Hydrogen Energy.

[15] Ishaq H., Dincer I., Crawford C. (2022). A review on hydrogen production and utilization: Challenges and opportunities. Int. J. Hydrogen Energy.

[16] Cho H.H., Strezov V., Evans T.J. (2023). A review on global warming potential, challenges and opportunities of renewable hydrogen production technologies. Sustain. Mater. Technol..

[17] Tahir M.B., Riaz K.N. (2021). Fundamentals of Photocatalysis for Energy Conversion. Nanomaterials and Photocatalysis in Chemistry.

[18] Zeeshan H.M., Sharma S., Panahi M., Voloshina E., Dedkov Y. (2022). Semiconducting eutectic materials for photocatalysis and photoelectrochemistry applications: A perspective. Phys. Chem. Chem. Phys..

[19] Li R. (2017). Latest progress in hydrogen production from solar water splitting via photocatalysis, photoelectrochemical, and photovoltaic-photoelectrochemical solutions. Chin. J. Catal..

[20] Sun W., Zhu J., Zhang M., Meng X., Chen M., Feng Y., Chen X., Ding Y. (2022). Recent advances and perspectives in cobalt-based heterogeneous catalysts for photocatalytic water splitting, CO_2_ reduction, and N_2_ fixation. Chin. J. Catal..

[21] Isaacs M., Garcia-Navarro J., Ong W.J., Jiménez-Calvo P. (2022). Is Photocatalysis the Next Technology to Produce Green Hydrogen to Enable the Net Zero Emissions Goal?. Glob. Chall..

[22] Fajrina N., Tahir M. (2019). A critical review in strategies to improve photocatalytic water splitting towards hydrogen production. Int. J. Hydrogen Energy.

[23] Wang G., Chang J., Tang W., Xie W., Ang Y.S. (2022). 2D materials and heterostructures for photocatalytic water-splitting: A theoretical perspective. J. Phys. D Appl. Phys..

[24] Wang Z., Wang L. (2018). Progress in designing effective photoelectrodes for solar water splitting. Cuihua Xuebao/Chin. J. Catal..

[25] Jiang C., Moniz S.J.A., Wang A., Zhang T., Tang J. (2017). Photoelectrochemical devices for solar water splitting—Materials and challenges. Chem. Soc. Rev..

[26] Joy J., Mathew J., George S.C. (2018). Nanomaterials for photoelectrochemical water splitting—Review. Int. J. Hydrogen Energy.

[27] Minggu L.J., Daud W.R.W., Kassim M.B. (2010). An overview of photocells and photoreactors for photoelectrochemical water splitting. Int. J. Hydrogen Energy.

[28] Bhatt M.D., Lee J.S. (2015). Recent theoretical progress in the development of photoanode materials for solar water splitting photoelectrochemical cells. J. Mater. Chem. A Mater..

[29] Jeong S.Y., Song J., Lee S. (2018). Photoelectrochemical Device Designs toward Practical Solar Water Splitting: A Review on the Recent Progress of BiVO_4_ and BiFeO_3_ Photoanodes. Appl. Sci..

[30] Wu H., Tan H.L., Toe C.Y., Scott J., Wang L., Amal R., Ng Y.H. (2020). Photocatalytic and Photoelectrochemical Systems: Similarities and Differences. Adv. Mater..

[31] Guo Z., Zhou J., Zhu L., Sun Z. (2016). MXene: A promising photocatalyst for water splitting. J. Mater. Chem. A Mater..

[32] Sharma P., Jang J.W., Lee J.S. (2019). Key Strategies to Advance the Photoelectrochemical Water Splitting Performance of α-Fe_2_O_3_ Photoanode. ChemCatChem.

[33] Seabold J.A., Neale N.R. (2015). All first row transition metal oxide photoanode for water splitting based on Cu_3_V_2_O_8_. Chem. Mater..

[34] Guo L.J., Luo J.W., He T., Wei S.H., Li S.S. (2018). Photocorrosion-Limited Maximum Efficiency of Solar Photoelectrochemical Water Splitting. Phys. Rev. Appl..

[35] Zheng G., Wang J., Liu H., Murugadoss V., Zu G., Che H., Lai C., Li H., Ding T., Gao Q. (2019). Tungsten oxide nanostructures and nanocomposites for photoelectrochemical water splitting. Nanoscale.

[36] Xu X., Zhou G., Dong X., Hu J. (2017). Interface Band Engineering Charge Transfer for 3D MoS_2_ Photoanode to Boost Photoelectrochemical Water Splitting. ACS Sustain. Chem. Eng..

[37] Wang J., Sun H., Huang J., Li Q., Yang J. (2014). Band Structure Tuning of TiO_2_ for Enhanced Photoelectrochemical Water Splitting. J. Phys. Chem. C.

[38] Lv R., Wang T., Su F., Zhang P., Li C., Gong J. (2014). Facile synthesis of ZnO nanopencil arrays for photoelectrochemical water splitting. Nano Energy.

[39] Rahman G., Joo O.S. (2012). Photoelectrochemical water splitting at nanostructured α-Fe_2_O_3_ electrodes. Int. J. Hydrogen Energy.

[40] Kalanur S.S., Duy L.T., Seo H. (2018). Recent Progress in Photoelectrochemical Water Splitting Activity of WO_3_ Photoanodes. Top. Catal..

[41] Yu Z., Liu H., Zhu M., Li Y., Li W. (2021). Interfacial Charge Transport in 1D TiO_2_ Based Photoelectrodes for Photoelectrochemical Water Splitting. Small.

[42] Muzakkar M.Z., Umar A.A., Ilham I., Saputra Z., Zulfikar L., Maulidiyah M., Wibowo D., Ruslan R., Nurdin M. (2019). Chalcogenide material as high photoelectrochemical performance Se doped TiO_2_/Ti electrode: Its application for Rhodamine B degradation. Journal of Physics: Conference Series.

[43] Ozawa K., Emori M., Yamamoto S., Yukawa R., Yamamoto S., Hobara R., Fujikawa K., Sakama H., Matsuda I. (2014). Electron-hole recombination time at TiO_2_ single-crystal surfaces: Influence of surface band bending. J. Phys. Chem. Lett..

[44] Wang Z., Huang H., Li G., Yan X., Yu Z., Wang K., Wu Y. (2021). Advances in engineering perovskite oxides for photochemical and photoelectrochemical water splitting. Applied Physics Reviews.

[45] Guerrero A., Bisquert J. (2017). Perovskite semiconductors for photoelectrochemical water splitting applications. Curr. Opin. Electrochem..

[46] Grinberg I., West D.V., Torres M., Gou G., Stein D.M., Wu L., Chen G., Gallo E.M., Akbashev A.R., Davies P.K. (2013). Perovskite oxides for visible-light-absorbing ferroelectric and photovoltaic materials. Nature.

[47] Young S.M., Rappe A.M. (2012). First principles calculation of the shift current photovoltaic effect in ferroelectrics. Phys. Rev. Lett..

[48] Li L., Salvador P.A., Rohrer G.S. (2014). Photocatalysts with internal electric fields. Nanoscale.

[49] Jung H.S., Park N.G. (2015). Perovskite Solar Cells: From Materials to Devices. Small.

[50] Sophocleous M. (2004). Global and regional water availability and demand: Prospects for the future. Nat. Resour. Res..

[51] Ahmed S., Rasul M.G., Martens W.N., Brown R., Hashib M.A. (2010). Advances in Heterogeneous Photocatalytic Degradation of Phenols and Dyes in Wastewater: A Review. Water Air Soil Pollut..

[52] Pandey A., Kumar R.R., Kalidasan B., Laghari I.A., Samykano M., Kothari R., Abusorrah A.M., Sharma K., Tyagi V. (2021). Utilization of solar energy for wastewater treatment: Challenges and progressive research trends. J. Environ. Manag..

[53] Al-Nuaim M.A., Alwasiti A.A., Shnain Z.Y. (2022). The photocatalytic process in the treatment of polluted water. Chem. Pap..

[54] Kanhere P., Chen Z. (2014). A Review on Visible Light Active Perovskite-Based Photocatalysts. Molecules.

[55] Li H., Zhu J., Wu Q., Zhuang J., Guo H., Ma Z., Ye Y. (2017). Enhanced photovoltaic properties of PbTiO_3_-based ferroelectric thin films prepared by a sol-gel process. Ceram. Int..

[56] Wani A.L., Ara A., Usmani J.A. (2015). Lead toxicity: A review. Interdiscip. Toxicol..

[57] Castillo M.E., Shvartsman V.V., Gobeljic D., Gao Y., Landers J., Wende H., Lupascu D.C. (2013). Effect of particle size on ferroelectric and magnetic properties of BiFeO_3_ nanopowders. Nanotechnology.

[58] Qiao X., Geng W., Sun Y., Zheng D., Yang Y., Meng J., He J., Bi K., Cui M., Chou X. (2021). Robust in-plane polarization switching in epitaxial BiFeO3 films. J. Alloys Compd..

[59] Deng J., Banerjee S., Mohapatra S.K., Smith Y.R., Misra M. (2011). Bismuth Iron Oxide Nanoparticles as Photocatalyst for Solar Hydrogen Generation from Water. J. Fundam. Renew. Energy Appl..

[60] Gao T., Chen Z., Zhu Y., Niu F., Huang Q., Qin L., Sun X., Huang Y. (2014). Synthesis of BiFeo3 nanoparticles for the visible-light induced photocatalytic property. Mater. Res. Bull..

[61] Wang N., Luo X., Han L., Zhang Z., Zhang R., Olin H., Yang Y. (2020). Structure, Performance, and Application of BiFeO_3_ Nanomaterials. Nano-Micro Lett..

[62] Qiao L., Zhang S., Xiao H.Y., Singh D.J., Zhang K.H.L., Liu Z.J., Zu X.T., Li S. (2018). Orbital controlled band gap engineering of tetragonal BiFeO_3_ for optoelectronic applications. J. Mater. Chem. C Mater..

[63] Shah J.H., Malik A.S., Idris A.M., Rasheed S., Han H., Li C. (2021). Intrinsic photocatalytic water oxidation activity of Mn-doped ferroelectric BiFeO_3_. Chin. J. Catal..

[64] Yun Q., Xing W., Chen J., Gao W., Bai Y., Zhao S. (2015). Effect of Ho and Mn co-doping on structural, ferroelectric and ferromagnetic properties of BiFeO_3_ thin films. Thin Solid Films.

[65] Preethi A.J., Ragam M. (2021). Effect of doping in multiferroic BFO: A review. J. Adv. Dielectr..

[66] Xian T., Yang H., Dai J.F., Wei Z.Q., Ma J.Y., Feng W.J. (2011). Photocatalytic properties of BiFeO_3_ nanoparticles with different sizes. Mater. Lett..

[67] Dhawan A., Sudhaik A., Raizada P., Thakur S., Ahamad T., Thakur P., Singh P., Hussain C.M. (2023). BiFeO_3_-based Z scheme photocatalytic systems: Advances, mechanism, and applications. J. Ind. Eng. Chem..

[68] Li S., Lin Y.H., Zhang B.P., Wang Y., Nan C.W. (2010). Controlled fabrication of BiFeO_3_ uniform microcrystals and their magnetic and photocatalytic behaviors. J. Phys. Chem. C.

[69] Gao F., Chen X.Y., Yin K.B., Dong S., Ren Z.F., Yuan F., Yu T., Zou Z.G., Liu J. (2007). Visible-Light Photocatalytic Properties of Weak Magnetic BiFeO_3_ Nanoparticles. Adv. Mater..

[70] Zhao C., Zhang H., Cheng X. (2022). Spectroscopic study on the valence state of Fe in BiFeO_3_. J. Solid State Chem..

[71] Arifiadi A.N., Kim K.T., Khairani I.Y., Park C.B., Kim K.H., Kim S.K. (2021). Synthesis and multiferroic properties of high-purity CoFe_2_O_4_–BiFeO_3_ nanocomposites. J. Alloys Compd..

[72] Benyoussef M., Saitzek S., Rajput N.S., Courty M., El Marssi M., Jouiad M. (2022). Experimental and Theoretical Investigations of Low-Dimensional BiFeO_3_ System for Photocatalytic Applications. Catalysts.

[73] Sando D., Carrétéro C., Grisolia M.N., Barthélémy A., Nagarajan V., Bibes M. (2018). Revisiting the Optical Band Gap in Epitaxial BiFeO_3_ Thin Films. Adv. Opt. Mater..

[74] Arazas A.P.R., Wu C.C., Chang K.S. (2018). Hydrothermal fabrication and analysis of piezotronic-related properties of BiFeO_3_ nanorods. Ceram. Int..

[75] Subhiksha V., Kokilavani S., Khan S.S. (2022). Recent advances in degradation of organic pollutant in aqueous solutions using bismuth based photocatalysts: A review. Chemosphere.

[76] Benyoussef M., Saitzek S., Rajput N.S., El Marssi M., Jouiad M. (2023). Effect of Sr and Ti substitutions on optical and photocatalytic properties of Bi1-xSrxFe1-xTixO_3_ nanomaterials. Nanoscale Adv..

[77] Zhou T., Zhai T., Shen H., Wang J., Min R., Ma K., Zhang G. (2023). Strategies for enhancing performance of perovskite bismuth ferrite photocatalysts (BiFeO_3_): A comprehensive review. Chemosphere.

[78] Liu L., Huang H. (2022). Ferroelectrics in Photocatalysis. Chem. A Eur. J..

[79] Zhu Q., Zhang K., Li D., Li N., Xu J., Bahnemann D.W., Wang C. (2021). Polarization-enhanced photocatalytic activity in non-centrosymmetric materials based photocatalysis: A review. Chem. Eng. J..

[80] Shvartsman V.V., Kleemann W., Haumont R., Kreisel J. (2007). Large bulk polarization and regular domain structure in ceramic BiFeO_3_. Appl. Phys. Lett..

[81] Lebeugle D., Colson D., Forget A., Viret M. (2007). Very large spontaneous electric polarization in BiFeO_3_ single crystals at room temperature and its evolution under cycling fields. Appl. Phys. Lett..

[82] Wang Y.P., Zhou L., Zhang M.F., Chen X.Y., Liu J.M., Liu Z.G. (2004). Room-temperature saturated ferroelectric polarization in BiFeO_3_ ceramics synthesized by rapid liquid phase sintering. Appl. Phys. Lett..

[83] Song S.H., Zhu Q.S., Weng L.Q., Mudinepalli V.R. (2015). A comparative study of dielectric, ferroelectric and magnetic properties of BiFeO_3_ multiferroic ceramics synthesized by conventional and spark plasma sintering techniques. J. Eur. Ceram. Soc..

[84] Wahba M.A., Yakout S.M., Youssef A.M., Sharmoukh W., Elsayed A.M., Khalil M.S. (2022). Chelating Agents Assisted Rapid Synthesis of High Purity BiFeO_3_: Remarkable Optical, Electrical, and Magnetic Characteristics. J. Supercond. Nov. Magn..

[85] Tahir M., Riaz S., Ahmad N., Khan U., Atiq S., Iqbal M.J., Naseem S. (2019). Anomalous dielectric behavior and correlation of barrier hopping mechanism with ferroelectricity in solvent assisted phase pure bismuth iron oxide nanoparticles. Mater. Res. Bull..

[86] Banoth P., Sohan A., Kandula C., Kollu P. (2022). Structural, dielectric, magnetic, and ferroelectric properties of bismuth ferrite (BiFeO_3_) synthesized by a solvothermal process using hexamethylenetetramine (HMTA) as precipitating agent. Ceram. Int..

[87] Teague J.R., Gerson R., James W.J. (1970). Dielectric hysteresis in single crystal BiFeO_3_. Solid State Commun..

[88] BaoLin F., Hao X., ZhaoXian X. (2010). Articles Structure and multiferroic properties of Y-doped BiFeO_3_ ceramics. Chin. Sci. Bull..

[89] Layek S., Verma H.C. (2015). Magnetic and Dielectric Properties of Multiferroic BiFeO_3_ Nanoparticles Synthesized by a Novel Citrate Combustion Method. Adv. Mater. Lett..

[90] Du Y., Cheng Z.X., Shahbazi M., Collings E.W., Dou S.X., Wang X.L. (2010). Enhancement of ferromagnetic and dielectric properties in lanthanum doped BiFeO_3_ by hydrothermal synthesis. J. Alloys Compd..

[91] Suresh P., Srinath S. (2014). A comprative study of sol-gel and solid-state prepared La^3+^ doped multiferroic BiFeO_3_. Adv. Mater. Lett..

[92] Zhang G.D., Dai J.Q., Liang X.L. (2023). Enhanced ferroelectric properties in La-doped BiFeO_3_ films by the sol-gel method. J. Sol-Gel Sci. Technol..

[93] Sheoran N., Kumar A., Kumar V., Banerjee A. (2020). Structural, Optical, and Multiferroic Properties of Yttrium (Y^3+^)-Substituted BiFeO_3_ Nanostructures. J. Supercond. Nov. Magn..

[94] Dhir G., Verma N.K. (2020). Correlation of spin, size and structure in sol-gel prepared doped BiFeO_3_ nanoparticles. J. Mol. Struct..

[95] Ozdilek C., Ozenbas M. (2020). Hydrothermal synthesis of Yb-doped BiFeO_3_ crystallites and their structural, magnetic and electrical properties. Ceram. Int..

[96] Suresh S., Kathirvel A., Maheswari A.U., Sivakumar M. (2019). Frequency dependent dielectric relaxation of Ba-doped BiFeO_3_ nanoparticles. Mater. Res. Express.

[97] Mazumder R., Sen A. (2009). Effect of Pb-doping on dielectric properties of BiFeO_3_ ceramics. J. Alloys Compd..

[98] Wrzesinska A., Khort A., Bobowska I., Busiakiewicz A., Wypych-Puszkarz A. (2019). Influence of the La^3+^, Eu^3+^, and Er^3+^ Doping on Structural, Optical, and Electrical Properties of BiFeO_3_ Nanoparticles Synthesized by Microwave-Assisted Solution Combustion Method. J. Nanomater..

[99] Rani S., Sanghi S., Agarwal A., Kumar R., Singh O. (2022). Crystal structure, magnetic and dielectric properties of Er-doped BiFeO_3_ ceramics. Appl. Phys. A Mater. Sci. Process..

[100] Shinjo Y., Mori M., Fujihara S., Hagiwara M. (2022). Ti doping and low-temperature sintering of BiFeO_3_ nanoparticles synthesized by the solvothermal method. Ceram. Int..

[101] Kathirvel A., Krishna K.N.I., Ganga R., Maheswari A.U., Sivakumar M. (2022). Enhanced magnetic, dielectric and photoconductive properties of Zr doped BiFeO_3_ nanostructures. Phys. E Low-Dimens. Syst. Nanostruct..

[102] Nadeem M., Khan W., Khan S., Husain S., Ansari A. (2018). Tailoring dielectric properties and multiferroic behavior of nanocrystalline BiFeO_3_ via Ni doping. J. Appl. Phys..

[103] Saxena P., Kumar A., Sharma P., Varshney D. (2016). Improved dielectric and ferroelectric properties of dual-site substituted rhombohedral structured BiFeO_3_ multiferroics. J. Alloys Compd..

[104] Godara S., Kumar B. (2015). Effect of Ba-Nb co-doping on the structural, dielectric, magnetic and ferroelectric properties of BiFeO_3_ nanoparticles. Ceram. Int..

[105] Xu D., Zhao W., Cao W., Li W., Fei W. (2021). Electrical properties of Li and Nb modified BiFeO_3_ ceramics with reduced leakage current. Ceram. Int..

[106] Sharif M.K., Khan M.A., Warsi M.F., Ramzan M., Hussain A. (2018). Structural and ferroelectric properties of hafnium substituted BiFeO_3_ multiferroics synthesized via auto combustion technique. Ceram. Int..

[107] Priyadharsini P., Pradeep A., Sathyamoorthy B., Chandrasekaran G. (2014). Enhanced multiferroic properties in la and Ce co-doped BiFeO_3_ nanoparticles. J. Phys. Chem. Solids.

[108] Sen S., Mondal A., Parida R.K., Parida B.N. (2022). Improved optical, dielectric, impedance, and magnetic properties of (BiFeO_3_)0.6(CaTiO_3_)0.4 for multifunctional utilities. Inorg. Chem. Commun..

[109] Baloni M., Sharma R.C., Singh H., Singh M.K., Kumar A., Sati P.C., Khan B., Thakur V.N. (2022). Effect of Nd doping on structural, dielectric, magnetic and ferroelectric properties of 0.8BiFeO_3_–0.2PbTiO_3_ solid solution. J. Alloys Compd..

[110] Ren Y., Liu H., Liu F., Liu G. (2021). Tuning of electric and magnetic properties of BiFeO_3_-SrTiO_3_ solid solution ceramics by site-specific doping of Mn. J. Alloys Compd..

[111] Ji C., Fan T., Chen G., Bai X., Wang J., He J., Cai W., Gao R., Deng X., Wang Z. (2021). Influence of sintering method on microstructure, electrical and magnetic properties of BiFeO_3_–BaTiO_3_ solid solution ceramics. Mater. Today Chem..

[112] Zia L., Jaffari G.H., Khan N.A., Rahman J.U., Lee S., Shah S.I. (2021). Identification and comparison of peculiarities in physical properties of multiferroic morphotrophic phase boundary sintered BiFeO_3_-xPbTiO_3_ nano-ceramics. J. Phys. Chem. Solids.

[113] Shankar S., Maurya I., Raj A., Singh S., Thakur O.P., Jayasimhadri M. (2020). Dielectric and tunable ferroelectric properties in BiFeO_3_–BiCoO_3_–BaTiO_3_ ternary compound. Appl. Phys. A Mater. Sci. Process..

[114] Zhang X., Yan J., Shi R., Wang Z., Zhang M., Du Q., Qi X. (2020). Structural, dielectric, and multiferroic properties of Ta_2_O_5_-modified BiFeO_3_–BaTiO_3_–LaFeO_3_ solid solutions. J. Mater. Sci. Mater. Electron..

[115] Tang Z., Zhuang J., Bokov A.A., Luo Z., Kubrin S.P., Raevski I.P., Ma M., Zhang N., Zhang J., Liu Z. (2021). Multiscale Domain Structures and Ferroic Properties of Dy-Modified BiFeO_3_-PbTiO_3_ Single Crystals. Cryst. Growth Des..

[116] Masso R., Tripathy S.N., Aponte F.A., Pradhan D.K., Martinez R., Palai R. (2021). Structural and magnetodielectric properties of BiFeO_3_-GdMnO_3_ multiferroics. Mater. Res. Express.

[117] Kumar N., Narayan B., Singh A.K., Kumar S. (2020). Enhanced magneto-capacitance in Sr^2+^ modified BiFeO_3_–PbTiO_3_ solid solutions. Mater. Chem. Phys..

[118] Praharaj S., Singha A., Rout D. (2021). Dielectric and piezoelectric properties of lead-free Na0.5Bi0.5TiO_3_-SrTiO_3_-BiFeO_3_ ternary system. J. Alloys Compd..

[119] Ponraj C., Vinitha G., Daniel J. (2020). Visible light photocatalytic activity of Mn-doped BiFeO_3_ nanoparticles. Int. J. Green Energy.

[120] Gao T., Chen Z., Huang Q., Niu F., Huang X., Qin L., Huang Y. (2015). A review: Preparation of bismuth ferrite nanoparticles and its applications in visible-light induced photocatalyses. Rev. Adv. Mater. Sci..

[121] Sharmin F., Basith M.A. (2022). Highly efficient photocatalytic degradation of hazardous industrial and pharmaceutical pollutants using gadolinium doped BiFeO_3_ nanoparticles. J. Alloys Compd..

[122] Mohan S., Subramanian B., Bhaumik I., Gupta P.K., Jaisankar S.N. (2014). Nanostructured Bi_(1−x)_Gd_(x)_FeO_3_—A multiferroic photocatalyst on its sunlight driven photocatalytic activity. RSC Adv..

[123] Shi J., Guo L. (2012). ABO_3_-based photocatalysts for water splitting. Prog. Nat. Sci. Mater. Int..

[124] Guo R., Fang L., Dong W., Zheng F., Shen M. (2010). Enhanced photocatalytic activity and ferromagnetism in Gd doped BiFeO_3_ nanoparticles. J. Phys. Chem. C.

[125] Vanga P.R., Mangalaraja R.V., Ashok M. (2015). Structural, magnetic and photocatalytic properties of La and alkaline co-doped BiFeO_3_ nanoparticles. Mater. Sci. Semicond. Process..

[126] Sakar M., Balakumar S., Saravanan P., Bharathkumar S. (2015). Compliments of confinements: Substitution and dimension induced magnetic origin and band-bending mediated photocatalytic enhancements in Bi1−xDyxFeO_3_ particulate and fiber nanostructures. Nanoscale.

[127] Zhou J., Jiang L., Chen D., Liang J., Qin L., Bai L., Sun X., Huang Y. (2019). Facile synthesis of Er-doped BiFeO_3_ nanoparticles for enhanced visible light photocatalytic degradation of tetracycline hydrochloride. J. Sol-Gel Sci. Technol..

[128] Chen Z., Wu Y., Wang X., Jin W., Zhu C. (2015). Ferromagnetism and enhanced photocatalytic activity in Nd doped BiFeO_3_ nanopowders. J. Mater. Sci. Mater. Electron..

[129] Hu Z., Chen D., Wang S., Zhang N., Qin L., Huang Y. (2017). Facile synthesis of Sm-doped BiFeO_3_ nanoparticles for enhanced visible light photocatalytic performance. Mater. Sci. Eng. B.

[130] Soltani T., Lee B.K. (2017). Comparison of benzene and toluene photodegradation under visible light irradiation by Ba-doped BiFeO_3_ magnetic nanoparticles with fast sonochemical synthesis. Photochem. Photobiol. Sci..

[131] Ponraj C., Kumar P.S., Sarkar S., Krishnamoorthi C., Manikandan N., Vinitha G., Daniel J. (2022). Enhanced visible light photocatalytic activity of magnetic cobalt doped BiFeO_3_. Surf. Interfaces.

[132] Jaffari Z.H., Lam S.M., Sin J.C., Zeng H., Mohamed A.R. (2020). Magnetically recoverable Pd-loaded BiFeO_3_ microcomposite with enhanced visible light photocatalytic performance for pollutant, bacterial and fungal elimination. Sep. Purif. Technol..

[133] Umar M., Mahmood N., Awan S.U., Fatima S., Mahmood A., Rizwan S. (2019). Rationally designed La and Se co-doped bismuth ferrites with controlled bandgap for visible light photocatalysis. RSC Adv..

[134] Kebede M.T., Devi S., Tripathi B., Chauhan S., Dillu V. (2022). Structural transition and enhanced magnetic, optical and photocatalytic properties of novel Ce–Ni co-doped BiFeO_3_ nanoparticles. Mater. Sci. Semicond. Process..

[135] Vanga P.R., Mangalaraja R.V., Ashok M. (2015). Effect of (Nd, Ni) co-doped on the multiferroic and photocatalytic properties of BiFeO_3_. Mater. Res. Bull..

[136] Soltani T., Tayyebi A., Lee B.K. (2020). BiFeO_3_/BiVO_4_ p−n heterojunction for efficient and stable photocatalytic and photoelectrochemical water splitting under visible-light irradiation. Catal. Today.

[137] Bargozideh S., Tasviri M., Kianifar M. (2020). Construction of novel magnetic BiFeO_3_/MoS_2_ composite for enhanced visible-light photocatalytic performance towards purification of dye pollutants. Int. J. Environ. Anal. Chem..

[138] Xu J., Qin T., Chen W., Lv J., Zeng X., Sun J., Li Y.-Y., Zhou J. (2021). Synergizing piezoelectric and plasmonic modulation of Ag/BiFeO_3_ fibrous heterostructure toward boosted photoelectrochemical energy conversion. Nano Energy.

[139] Ghorbani M., Sheibani S., Abdizadeh H., Golobostanfard M.R. (2023). Modified BiFeO_3_/rGO nanocomposite by controlled synthesis to enhance adsorption and visible-light photocatalytic activity. J. Mater. Res. Technol..

[140] Wang X., He X.-S., Li C.-Y., Liu S.-L., Lu W., Xiang Z., Wang Y. (2023). Sonocatalytic removal of tetracycline in the presence of S-scheme Cu_2_O/BiFeO_3_ heterojunction: Operating parameters, mechanisms, degradation pathways and toxicological evaluation. J. Water Process. Eng..

[141] Wang Y., Tang Y., Sun J., Wu X., Liang H., Qu Y., Jing L. (2022). BiFeO_3_/Bi_2_Fe_4_O_9_ S-scheme heterojunction hollow nanospheres for high-efficiency photocatalytic o-chlorophenol degradation. Appl. Catal. B Environ..

[142] Marwat M.A., Ullah H., Usman M., Ehsan M.A., Zhang H., Khan M.F., Ali S., Yousaf M. (2022). Significantly improved photocatalytic activity of the SnO_2_/BiFeO_3_ heterojunction for pollutant degradation and mechanism. Ceram. Int..

[143] Subramanian Y., Ramasamy V., Karthikeyan R., Srinivasan G.R., Arulmozhi D., Gubendiran R.K., Sriramalu M. (2019). Investigations on the enhanced dye degradation activity of heterogeneous BiFeO_3_–GdFeO_3_ nanocomposite photocatalyst. Heliyon.

[144] Niu F., Chen D., Qin L., Zhang N., Wang J., Chen Z., Huang Y. (2015). Facile Synthesis of Highly Efficient p–n Heterojunction CuO/BiFeO_3_ Composite Photocatalysts with Enhanced Visible-Light Photocatalytic Activity. ChemCatChem.

[145] Ghasemi A., Hasheminiasari M., Masoudpanah S.M., Safizade B. (2018). Enhanced Photocatalytic Activity of Two-Pot-Synthesized BiFeO_3_–ZnFe_2_O_4_ Heterojunction Nanocomposite. J. Electron. Mater..

[146] Liao X., Li T.-T., Ren H.-T., Mao Z., Zhang X., Lin J.-H., Lou C.-W. (2021). Enhanced photocatalytic performance through the ferroelectric synergistic effect of p-n heterojunction BiFeO_3_/TiO_2_ under visible-light irradiation. Ceram. Int..

[147] Banoth P., Narsaiah B.P., De Los Santos Valladares L., Kargin J., Kollu P. (2023). Single-phase BiFeO_3_ and BiFeO_3_–Fe2O_3_ nanocomposite photocatalysts for photodegradation of organic dye pollutants. Nanoscale Adv..

[148] Ramezanalizadeh H., Manteghi F. (2017). Design and development of a novel BiFeO_3_/CuWO_4_ heterojunction with enhanced photocatalytic performance for the degradation of organic dyes. J. Photochem. Photobiol. A Chem..

[149] Tran V.T., Chen D.H. (2023). Ag_2_O@BiFeO_3_ heterostructure composite coupling built-in electric field with piezopotential for enhanced photocatalytic pollutant degradation and photoelectrochemical water splitting. Appl. Surf. Sci..

[150] Cui H., Wang Z., Cao G., Wu Y., Song J., Li Y., Zhang L., Mu J., Chou X. (2022). Facilitated Photocatalytic Degradation of Rhodamine B over One-Step Synthesized Honeycomb-Like BiFeO_3_/g-C_3_N_4_ Catalyst. Nanomaterials.

[151] Maeda K. (2011). Photocatalytic water splitting using semiconductor particles: History and recent developments. J. Photochem. Photobiol. C Photochem. Rev..

[152] Qi J., Liu H., Feng M., Xu H., Liu H., Wang C., Wang A., Lü W. (2020). Enhanced hydrogen evolution reaction in Sr doped BiFeO_3_ by achieving the coexistence of ferroelectricity and ferromagnetism at room temperature. J. Energy Chem..

[153] Man S., Leng X., Bai J., Kan S., Cui Y., Wang J., Xu L. (2023). Enhancement of photoelectrochemical performance of BiFeO_3_ by Sm^3+^ doping. Ceram. Int..

[154] Vishwakarma A.K., Tripathi P., Srivastava A., Sinha A.S.K., Srivastava O.N. (2017). Band gap engineering of Gd and Co doped BiFeO_3_ and their application in hydrogen production through photoelectrochemical route. Int. J. Hydrogen Energy.

[155] Khoomortezaei S., Abdizadeh H., Golobostanfard M.R. (2021). Ferro-photocatalytic Enhancement of Photoelectrochemical Water Splitting Using the WO_3_/BiFeO_3_ Heterojunction. Energy Fuels.

[156] Wu X., Li H., Wang X., Jiang L., Xi J., Du G., Ji Z. (2019). Ferroelectric enhanced photoelectrochemical water splitting in BiFeO_3_/TiO_2_ composite photoanode. J. Alloys Compd..

[157] Zhang T., Shen Y., Qiu Y., Liu Y., Xiong R., Shi J., Wei J. (2017). Facial Synthesis and Photoreaction Mechanism of BiFeO_3_/Bi_2_Fe_4_O_9_ Heterojunction Nanofibers. ACS Sustain. Chem. Eng..

[158] Yan X., Pu R., Xie R., Zhang B., Shi Y., Liu W., Ma G., Yang N. (2021). Design and fabrication of Bi_2_O_3_/BiFeO_3_ heterojunction film with improved photoelectrochemical performance. Appl. Surf. Sci..

[159] Bera S., Ghosh S., Shyamal S., Bhattacharya C., Basu R.N. (2019). Photocatalytic hydrogen generation using gold decorated BiFeO_3_ heterostructures as an efficient catalyst under visible light irradiation. Sol. Energy Mater. Sol. Cells.

[160] Kolivand A., Sharifnia S. (2021). Enhanced photocatalytic hydrogen evolution from water splitting by Z-scheme CdS/BiFeO_3_ heterojunction without using sacrificial agent. Int. J. Energy Res..

[161] Khoomortezaei S., Abdizadeh H., Golobostanfard M.R. (2019). Triple Layer Heterojunction WO_3_/BiVO_4_/BiFeO_3_ Porous Photoanode for Efficient Photoelectrochemical Water Splitting. ACS Appl. Energy Mater..

[162] Zhu J., He Y., Yang Y., Liu Y., Chen M., Cao D. (2021). BiFeO_3_/Cu_2_O Heterojunction for Efficient Photoelectrochemical Water Splitting Under Visible-Light Irradiation. Catal. Lett..

[163] Zhang P., Wang T., Gong J. (2015). Mechanistic Understanding of the Plasmonic Enhancement for Solar Water Splitting. Adv. Mater..

[164] Ghosh S., Mallik A.K., Basu R.N. (2018). Enhanced photocatalytic activity and photoresponse of poly(3,4-ethylenedioxythiophene) nanofibers decorated with gold nanoparticle under visible light. Sol. Energy.

[165] Tiburcio J., Sacari E., Chacaltana J., Medina J., Gamarra F., Polo C., Mamani E., Quispe A. (2023). Influence of Cr Doping on Structural, Optical, and Photovoltaic Properties of BiFeO_3_ Synthesized by Sol-Gel Method. Energies.

[166] Anjum N., Lamia S.N.E., Arafat M.Y., Mahboob M., Basith M.A. (2018). Photocatalytic properties of Ti-doped BiFeO_3_ bulk and nanoparticles for solar hydrogen fuel generation. AIP Conference Proceedings.

[167] Haydous F., Scarisoreanu N.D., Birjega R., Ion V., Lippert T., Dumitrescu N., Moldovan A., Andrei A., Teodorescu V.S., Ghica C. (2018). Rolling dopant and strain in Y-doped BiFeO_3_ epitaxial thin films for photoelectrochemical water splitting. Sci. Rep..

[168] Xu M., Lu M., Qin G., Wu X., Yu T., Zhang L., Li K., Cheng X., Lan Y. (2022). Piezo-Photocatalytic Synergy in BiFeO_3_@COF Z-Scheme Heterostructures for High-Efficiency Overall Water Splitting. Angew. Chem. Int. Ed..

[169] Sepahvand H., Sharifnia S. (2019). Photocatalytic overall water splitting by Z-scheme g-C_3_N_4_/BiFeO_3_ heterojunction. Int. J. Hydrogen Energy.

[170] Arif N., Ma Y., Iqbal M.A., Zafar M.N., Liang H., Zhang Q., Zeng Y.-J. (2023). Enhanced charge separation in dual Z-scheme Au decorated LaFeO_3_-g-C_3_N_4_-BiFeO_3_ system for efficient H_2_ production. Fuel.

[171] Zhang J., Zhang Y., Li L., Yan W., Wang H., Mao W., Cui Y., Li Y., Zhu X. (2023). Synergizing the internal electric field and ferroelectric polarization of the BiFeO_3_/ZnIn_2_S_4_ Z-scheme heterojunction for photocatalytic overall water splitting. J. Mater. Chem. A.

